# The p53/p73 - p21^CIP1^ tumor suppressor axis guards against chromosomal instability by restraining CDK1 in human cancer cells

**DOI:** 10.1038/s41388-020-01524-4

**Published:** 2020-11-09

**Authors:** Ann-Kathrin Schmidt, Karoline Pudelko, Jan-Eric Boekenkamp, Katharina Berger, Maik Kschischo, Holger Bastians

**Affiliations:** 1 Georg-August University Göttingen, Göttingen Center for Molecular Biosciences (GZMB) and University Medical Center Göttingen (UMG), Institute of Molecular Oncology, Section for Cellular Oncology, D-37077 Göttingen, Germany; 2 University of Applied Sciences Koblenz, Department of Mathematics and Technology, D-53424 Remagen, Germany

**Keywords:** Cancer genomics, Mitosis

## Abstract

Whole chromosome instability (W-CIN) is a hallmark of human cancer and contributes to the evolvement of aneuploidy. W-CIN can be induced by abnormally increased microtubule plus end assembly rates during mitosis leading to the generation of lagging chromosomes during anaphase as a major form of mitotic errors in human cancer cells. Here, we show that loss of the tumor suppressor genes *TP53* and *TP73* can trigger increased mitotic microtubule assembly rates, lagging chromosomes, and W-CIN. *CDKN1A*, encoding for the CDK inhibitor p21^CIP1^, represents a critical target gene of p53/p73. Loss of p21^CIP1^ unleashes CDK1 activity which causes W-CIN in otherwise chromosomally stable cancer cells. Consequently, induction of CDK1 is sufficient to induce abnormal microtubule assembly rates and W-CIN. *Vice versa*, partial inhibition of CDK1 activity in chromosomally unstable cancer cells corrects abnormal microtubule behavior and suppresses W-CIN. Thus, our study shows that the p53/p73 - p21^CIP1^ tumor suppressor axis, whose loss is associated with W-CIN in human cancer, safeguards against chromosome missegregation and aneuploidy by preventing abnormally increased CDK1 activity.

## Introduction

**Fig. 1:**
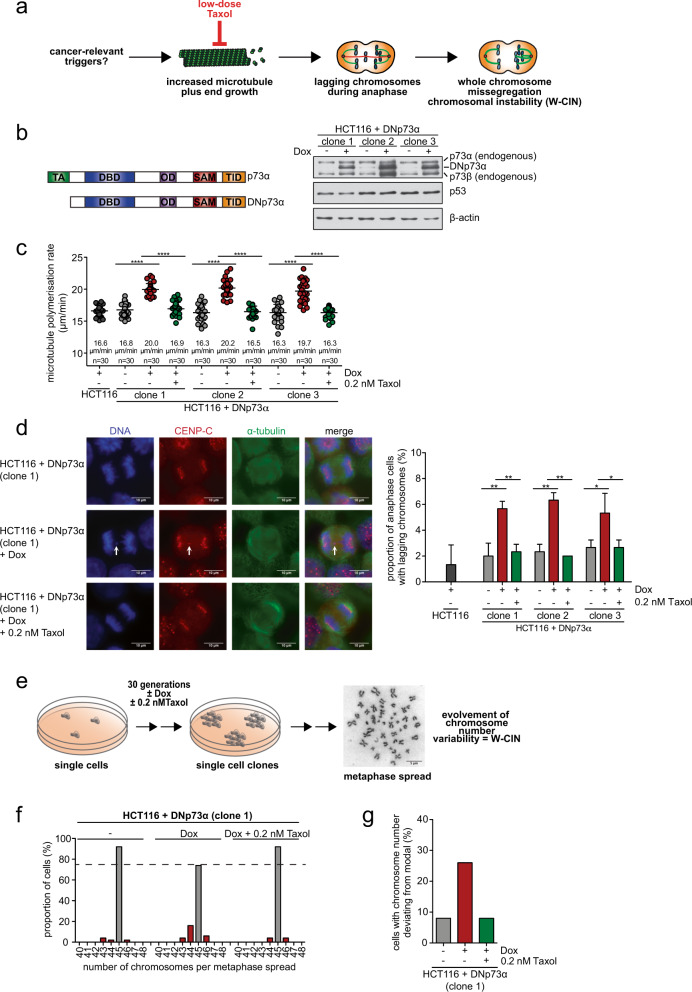
Expression of oncogenic *DNp73* induces whole chromosome instability by increasing mitotic microtubule plus end assembly rates. **a** Model depicting the causal relationship between increased microtubule plus end growth rates in mitosis and the induction of chromosome missegregation constituting W-CIN. **b** Left panel: Model for the structure of p73 and DNp73: transactivation domain (TA), DNA-binding domain (DBD), oligomerization domain (OD), SAM domain (SAM), transactivation inhibitory domain (TID). Right panel: Doxycycline-inducible expression of *DNp73* in chromosomally stable HCT116 cells. Three independent stable cell lines were treated with doxycycline for 24 h and expression of DNp73 was detected by western blotting. A representative western blot is shown. Note that the two bands for endogenous p73 represent the α and β isoforms of the protein. **c** Mitotic microtubule plus end assembly rates upon expression of *DNp73*. HCT116-DNp73 cell lines were treated with or without doxycycline and additionally with low-dose Taxol. As a control HCT116 parental cells were treated with doxycycline. Microtubule growth rates were determined by tracking EB3-GFP comets in mitotic cells by live-cell microscopy. Scatter dot plots show average microtubule polymerization rates (20 microtubules/cell, *n* = 30 mitotic cells from 3 independent experiments, mean ± SD, *t-*test). **d** Proportion of cells exhibiting lagging chromosomes in anaphase after expression of *DNp73* and after restoration of proper microtubule growth rates. HCT116 parental cells were incubated with doxycycline as a control. Left: Representative examples of anaphase cells with or without lagging chromosomes (white arrows), Scale bar, 10 µm. Right: The graph shows the proportion of anaphase cells exhibiting lagging chromosomes for three independent stable cell lines (*n* = 300 anaphase cells from three independent experiments, mean ± SD, *t*-test). **e** Scheme depicting the generation of single-cell clones in order to determine chromosome number variability as a measure of W-CIN. **f** Proportion of cells harboring the indicated chromosome numbers per cell (*n* = 50 metaphase spreads). **g** Proportion of cells with chromosome numbers deviating from the modal (45 chromosomes in HCT116 cells). The calculation is based on analyses of chromosome number variability shown in **f**.

Chromosomal instability (CIN) is a major form of genome instability representing a hallmark of cancer [1, 2]. While structural alterations are caused by structural chromosome instability (S-CIN), changes in the chromosome numbers, referred to as aneuploidy, are a result of whole chromosome instability (W-CIN). A majority of cancer cells shows aneuploidy that is strongly associated with tumorigenesis, tumor progression, therapy resistance, and poor prognosis [3]. W-CIN is defined by an elevated rate of perpetual missegregation of whole chromosomes or large parts thereof during mitosis [4]. An important type of mitotic errors contributing to whole chromosome missegregation in human cancer cells are so-called lagging chromosomes that are caused by unresolved merotelic microtubule-kinetochore attachments [5]. Various abnormalities in chromosomally unstable cancer cells can contribute to the generation of lagging chromosomes including supernumerary centrosomes [6, 7] or hyper-stable microtubule-kinetochore attachments [8, 9]. More recently, we have demonstrated that abnormally increased microtubule plus end assembly rates within mitotic spindles result in the induction of W-CIN in cancer cells [10–16]. In fact, increased microtubule assembly rates cause merotelic microtubule-kinetochore attachments, lagging chromosomes, and chromosome missegregation [10] (Fig. 1a).

Despite the strong link between increased mitotic microtubule assembly rates and W-CIN, the cancer-associated genetic alterations leading to abnormal microtubule dynamics are not well understood. So far, only a few tumor suppressors and oncogenes including *BRCA1*, *CHK2*, *AURKA*, and *CEP72* were shown to be involved in triggering W-CIN by increasing mitotic microtubule assembly rates [10–15]. Interestingly, loss of the tumor suppressor gene *TP53* is strongly associated with the presence of W-CIN [17]. However, loss of p53 alone is neither sufficient to trigger abnormal microtubule dynamics nor chromosome missegregation and W-CIN [10, 18]. p53 belongs to a family of transcription factors that also includes p73 [19]. While *TP53* is often mutated or deleted in cancer cells, *TP73* is infrequently mutated, and thus, it is still under debate whether *TP73* represents a tumor suppressor gene like *TP53* [20]. However, a truncated oncogenic form of p73, *DNp73*, is frequently overexpressed in various tumor types and can act as a dominant-negative regulator for both p73 and p53 [21, 22]. It is well established that both transcription factors have overlapping target genes including *CDKN1A*, which encodes for the cyclin-dependent kinase (CDK) inhibitor protein p21^CIP1^. p21^CIP1^ was shown to inhibit CDK1 and CDK2, thereby contributing to cell cycle arrest in G1 and G2 phase of the cell cycle, e.g., upon DNA damage [23] [24]. Interestingly, loss of p21^CIP1^ in unperturbed cells was shown to cause problems in chromosome segregation, which might reflect a requirement for p21^CIP1^ in fine-tuning mitotic progression [25]. In cancer, *CDKN1A* expression is often decreased, partly along with p53 inactivation, and associated with poor prognosis suggesting a possible tumor-suppressive function of *CDKN1A* [26].

Here, we provide a link between loss of the p53/p73 - p21^CIP1^ axis, mildly increased CDK1 activity and increased mitotic microtubule assembly rates and W-CIN.

## Material and methods

### Cell culture

HCT116, HT29, RKO, SW480, and SW620 cells were purchased from ATCC (USA), DLD-1 cells were from Sigma-Aldrich (Germany). DLD-1-*CDKN1A*^−/−^ cells were obtained from Horizon Discovery (UK). HCT116-*TP53*^−/−^ [27] and HCT116-*CDKN1A*^−/−^ cells [28] were kindly provided by Bert Vogelstein (Johns Hopkins University, Baltimore, USA). RKO cells expressing *CDKN1A* in a ponasterone A-inducible manner (RKO-p21-Pon) were kindly provided by Matthias Schmidt [29]. Details on culturing conditions are given in the Supplemental Methods.

### Measurement of microtubule plus end assembly rates

To determine microtubule plus end assembly rates, live-cell microscopy was performed on cells expressing GFP-tagged end-binding protein 3 (EB3) [10, 30] as described [11].

### Detection of lagging chromosomes

To quantify anaphase cells exhibiting lagging chromosomes, cells were fixed, stained, and imaged as described [15].

### W-CIN analysis

Single-cell clones were generated to determine the evolved chromosome number variability as a measure of W-CIN. Chromosome numbers were determined from metaphase spreads as described [10]. Details are given in the Supplemental Methods.

### Tumor data and bioinformatic analysis

Tumor data from the colon adenocarcinoma (COAD) and breast invasive carcinoma (BRCA) projects of The Cancer Genome Atlas (TCGA) program were obtained from the harmonized NCI Genomic Data Commons (GDC) database. We used Single Nucleotide Variants (SNVs) from whole-exome sequencing (Mutect2 Annotation), Copy Number Variations (CNVs) from Affymetrix 6.0 Genotyping Arrays and gene expression quantifications from high-throughput RNA sequencing. 389 COAD and 972 BRCA samples, for which all three data modalities were available, were used for further analyses. Additionally, RNA sequencing data of 113 BRCA and 41 COAD normal tissue samples were downloaded from the database.

For tumor transcriptome analysis, RNA sequencing count data were transformed into log2-counts per million (logCPM) expression values utilizing mean-variance modeling at the observational level (voom) [31] as implemented and described in the R-package limma [32]. These logCPM were centered around the mean logCPM of normal tissue samples for each gene respectively. The resulting differences were interpreted as the log2 fold change of gene expression in a tumor sample relative to normal tissue. To find differentially expressed genes, we used Wilcoxon’s rank sum test. To quantify chromosomal instability in the tumor samples, the weighted genome instability index score (wGII) was calculated using the SNP array-based copy-number variation data as described previously [33].

Classification of tumor samples: A sample is labeled as being mutated in a gene when a non-silent nucleotide variation can be observed in the SNV data. To divide the tumor samples into a lowCIN and highCIN group we used euclidean-distance average-linkage hierarchal clustering of the wGII. A sample was considered to exhibit low expression of a gene if the difference between its logCPM in that sample and the mean logCPM in normal samples is lower than −1. This is equivalent to a ratio between CPM in the tumor and (geometric) average CPM in normal tissue lower than half.

### Statistical analysis

For statistical analysis, the Graph Pad Prism 5.0 software (Graph Pad Software, USA) was used. Mean values and standard deviation (SD) were calculated. To analyze statistical significance, unpaired two-tailed *t*-tests (SD ≠ 0) or one-sample *t*-tests (SD = 0) were performed. *P* values were indicated as: *p < 0.05, **p < 0.01, ***p < 0.001, ****p < 0.0001, ns (not significant) *p* ≥ 0.05.

## Results

### Expression of oncogenic *DNp73* induces increased mitotic microtubule growth rates, chromosome missegregation, and W-CIN

We performed a mini-screen to identify cancer-relevant alterations that might contribute to an increase in mitotic microtubule growth rates and to the development of W-CIN and identified a truncated oncogenic form of p73 (*DNp73*) [22] (Fig. 1b, scheme). We generated HCT116 cell lines that express *DNp73* in a doxycycline (Dox) inducible manner (Fig. 1b) and measured microtubule growth rates in mitotic cells by tracking GFP-tagged microtubule end-binding protein 3 (EB3-GFP) by live-cell microscopy. Dox-induced expression of *DNp73* was sufficient to increase mitotic microtubule growth rates to a level typically observed in chromosomally unstable cancer cells (Fig. 1c) [10]. Moreover, *DNp73* expression caused the induction of lagging chromosomes during anaphase (Fig. 1d). Correction of abnormal microtubule growth rates by low-dose Taxol treatment (Fig. 1c) efficiently suppressed the generation of lagging chromosomes (Fig. 1d) indicating a causal relationship between both defects in the context of *DNp73* expression. Next, we investigated whether *DNp73* expression causes W-CIN in a microtubule growth rate-dependent manner. We evolved single-cell clones from uninduced (-Dox) or induced (+Dox) cells in the absence or presence of Taxol treatment (Fig. 1e). Karyotypic analyses of the otherwise chromosomally stable HCT116 cells revealed an induction of high chromosome number variability in cells with *DNp73* expression, which was suppressed upon correction of abnormal microtubule growth rates by Taxol (Fig. 1f, g). Thus, expression of *DNp73* acts as a *bona fide* inducer for abnormal mitotic microtubule assembly rates and W-CIN.

### Concomitant loss of p53 and p73 mimics expression of *DNp73* to induce an increase of microtubule growth rates and W-CIN

**Fig. 2:**
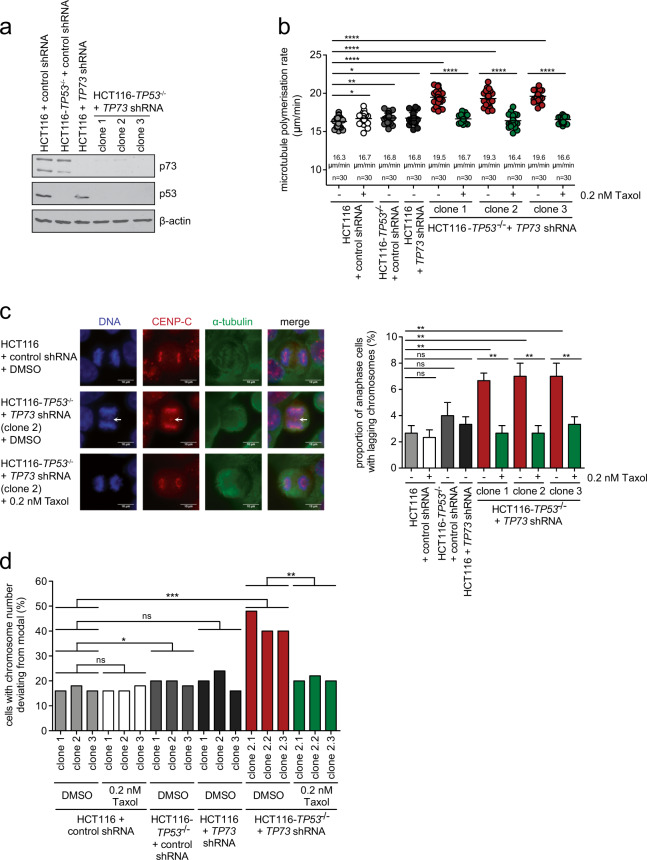
Concomitant loss of p53 and p73 induces chromosomal instability by increasing mitotic microtubule plus end assembly rates. **a** Generation of stable cell lines with loss of p53, p73 or concomitant loss of both. A representative western blot detecting p53 and p73 protein levels is shown. **b** Mitotic microtubule plus end assembly rates upon loss of p53, p73 or both. The indicated stable cell lines were used to measure microtubule plus end growth rates in mitosis by tracking EB3-GFP comets by live-cell microscopy. To restore normal microtubule growth rates, cells were treated with 0.2 nM Taxol for 16 h before measurement. Scatter dot plots show average microtubule polymerization rates (20 microtubules/cell, *n* = 30 mitotic cells from three independent experiments, mean ± SD, *t*-test). **c** Proportion of cells exhibiting lagging chromosomes in anaphase after loss of p53, p73 or both, and after restoration of proper microtubule growth rates. Left panel: Representative examples of anaphase cells with or without lagging chromosomes (white arrows), Scale bar, 10 µm. Right panel: The graph shows the proportion of anaphase cells exhibiting lagging chromosomes (*n* = 300 anaphase cells from 3 independent experiments, mean ± SD, *t*-test). **d** Induction of aneuploidy upon loss of p53/p73 and rescue upon restoration of proper microtubule growth rates. Single-cell clones derived from the indicated cell lines were grown in the presence or absence of 0.2 nM Taxol for 30 generations, chromosome numbers per cell were determined. The graph depicts the proportion of cells harboring chromosome numbers deviating from the modal (45 chromosomes in HCT116 cells, *n* = 50 cells).

DNp73 represents an oncogenic N-terminally truncated form of p73 lacking its transactivation domain that can act as a dominant-negative regulator for both p53 and p73 [22] (Fig. 1b, scheme). To test whether *DNp73* expression influences mitosis through inactivation of p53 and p73, we generated stable HCT116 cell lines with either single loss of p53, p73, or concomitant loss of both (Fig. 2a). As shown previously [10], loss of p53 alone was not sufficient to induce increased microtubule assembly rates (Fig. 2b) or lagging chromosomes (Fig. 2c). The same was observed upon single loss of p73. However, concomitant loss of p53 and p73 clearly increased microtubule growth rates to a level observed after *DNp73* expression (Fig. 2b). This was also associated with the induction of lagging chromosomes in anaphase (Fig. 2c). Rescue experiments using Taxol treatment showed that increased microtubule growth rates triggered by p53/p73 loss are responsible for whole chromosome missegregation (Fig. 2b, c). This was further supported by additional experiments, in which proper microtubule assembly rates were restored in p53/p73 deficient cells by partial downregulation of the microtubule plus end polymerase ch-TOG (encoded by *CKAP5*; Fig. S1a, S1b) [10], which led to suppression of lagging chromosomes (Fig. S1c).

To investigate whether concomitant loss of p53 and p73 induces W-CIN, we generated single-cell clones derived from the different *TP53*, *TP73*, or *TP53/TP73*-deficient cell lines and grew the cells in the presence or absence of Taxol for 30 generations. Analysis of chromosome number variability as a measure of W-CIN demonstrated the induction of aneuploidy over time only in cells with concomitant loss of p53 and p73, but not in cells with single loss of p53 or p73. W-CIN was efficiently suppressed upon correction of abnormal microtubule assembly rates by Taxol (Fig. 2d, Fig. S2), emphasizing again the causality between abnormal microtubule growth rates and W-CIN. Thus, concomitant loss of the transcription factors p53 and p73 mimics the mitotic defects observed upon *DNp73* expression and is sufficient to induce W-CIN.

### Loss of *CDKN1A* expression in response to loss of p53/p73 is crucial for the induction of increased microtubule growth rates and W-CIN

**Fig. 3:**
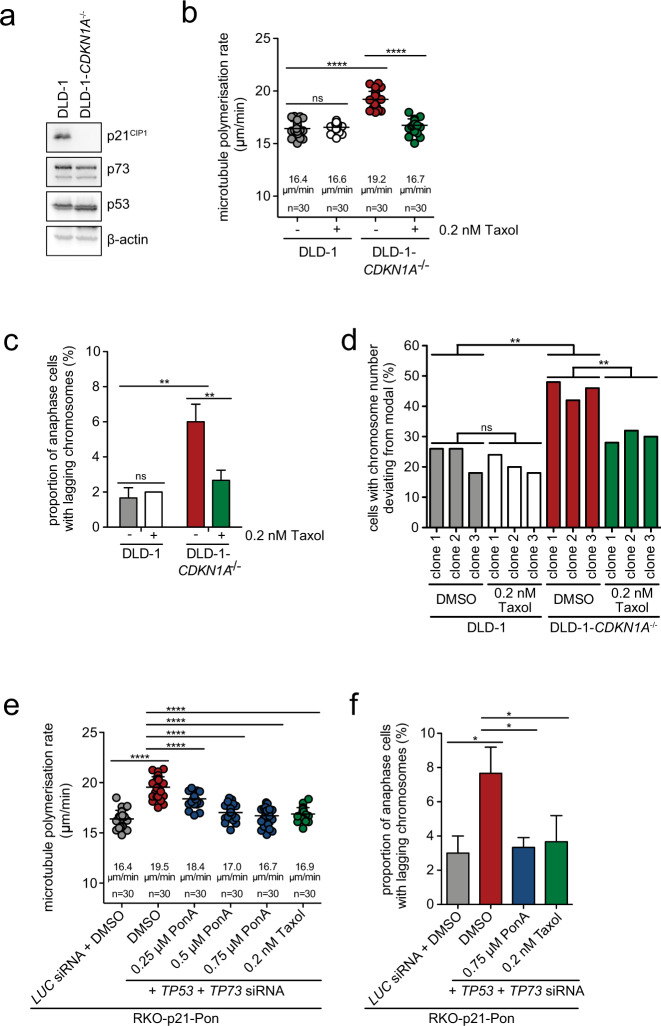
Increased microtubule growth rates and chromosomal instability after concomitant loss of p53 and p73 is mediated by loss of p21^CIP1^. **a** p73, p53, and p21^CIP1^ protein levels in chromosomally stable DLD-1 and DLD-1-*CDKN1A*^−/−^ cells. A representative western blot is shown. **b** Microtubule plus end assembly rates in mitotic DLD-1 and DLD-1-*CDKN1A*^−/−^ cells in the absence or presence of low-dose Taxol. Average microtubule polymerization rates are shown in the scatter dot plots. (20 microtubules/cell, *n* = 30 mitotic cells from three independent experiments, mean ± SD, *t*-test). **c** Proportion of cells exhibiting lagging chromosomes in anaphase after loss of p21^CIP1^, and after restoration of proper microtubule growth rates. The proportion of anaphase cells exhibiting lagging chromosomes was determined using the indicated cell lines (n = 300 anaphase cells from 3 independent experiments, mean ± SD, *t*-test). **d** Aneuploidy induction upon loss of *CDKN1A* expression. Single-cell clones derived from parental DLD-1 and DLD-1-*CDKN1A*^−/−^ cells were grown in the presence or absence of 0.2 nM Taxol for 30 generations, chromosome numbers per cell were determined. The graph shows the proportion of cells harboring chromosome numbers deviating from the modal (46 chromosomes in DLD-1 cells; *n* = 50 cells). **e** Microtubule plus end assembly rates in mitotic RKO-p21-Pon cells upon knockdown of *TP53* and *TP73* and ponasterone-A-induced re-expression of *CDKN1A*. Scatter dot plots show average microtubule polymerization rates (20 microtubules/cell, *n* = 30 mitotic cells from three independent experiments, mean ± SD, *t*-test). **f** Proportion of cells exhibiting lagging chromosomes in anaphase after loss of *TP53* and *TP73* and re-expression of *CDKN1A*. As control, cells were treated with low-dose Taxol to restore proper microtubule growth rates independently of *CDKN1A* expression (*n* = 300 anaphase cells from 3 independent experiments, mean ± SD, *t*-test).

It is well established that the transcription factors p53 and p73 have overlapping gene targets [34]. One of the premier targets of p53 and p73 is *CDKN1A*, which encodes for the cyclin-dependent kinase (CDK) inhibitor protein p21^CIP1^. p21^CIP1^ levels are strongly induced e.g., after DNA damage, inhibit CDK activities and thereby, mediate cell cycle arrest [27]. In unstressed cells, the levels of p21^CIP1^ are low and the role of p21^CIP1^ during an unperturbed cell cycle is less clear. We investigated if loss of p21^CIP1^ in response to loss of p53/p73 mediates the abnormal increase in microtubule growth rates and chromosome missegregation. We used chromosomally stable DLD-1 cells with or without homozygous deletion of *CDKN1A* (DLD-1-*CDKN1A*^−/−^; Fig. 3a). Indeed, loss of *CDKN1A* caused the same increase in microtubule growth rates during mitosis (Fig. 3b) and induced the generation of lagging chromosomes in anaphase in a microtubule growth rate-dependent manner (Fig. 3c). Experiments with HCT116 cells harboring a homozygous knockout for *CDKN1A* (Fig. S3a) verified these findings (Fig. S3b). Moreover, acute siRNA-mediated depletion of *TP53* and *TP73* resulted in significant downregulation of p21^CIP1^ levels (Fig. S3c) and was associated with an increase in mitotic microtubule growth rates (Fig. S3d). Karyotype analyses of single-cell clones derived from DLD-1-*CDKN1A*^−/−^ cells that were grown for 30 generations revealed the induction of W-CIN upon loss of *CDKN1A* expression, which was dependent on increased microtubule assembly rates (Fig. 3d, Fig. S3e, S3f). Hence, the loss of *CDKN1A* expression fully mimics the loss of p53/p73.

To further support a causal relationship between loss of p53/p73-p21^CIP1^ and mitotic defects leading to W-CIN, we re-expressed *CDKN1A* in cells with p53/p73 depletion. To avoid cell cycle arrest after high expression of *CDKN1A*, we employed a previously established chromosomally stable RKO cell line [29], in which *CDKN1A* is expressed in a ponasterone A-inducible manner (RKO-p21-Pon cells; Fig. S4a). As shown before [24, 35, 36], high levels of *CDKN1A* expression caused cell cycle arrest in G1 and G2 (Fig. S4b). RKO cells exhibited normal microtubule assembly rates and low levels of lagging chromosomes, both of which were induced upon p53/p73 depletion (Fig. 3e, f). Importantly, low-level expression of *CDKN1A*, which did not influence cell cycle progression (Fig. S4b), restored proper microtubule growth rates (Fig. 3e) and suppressed lagging chromosomes (Fig. 3f). Hence, mild re-expression of *CDKN1A* downstream of p53/p73 loss rescues the mitotic defects and chromosome missegregation indicating that p21^CIP1^ acts as an important mediator of the W-CIN driving defects.

### Loss of the p53/p73 – p21^CIP1^ axis unleashes CDK1 activity that mediates increased microtubule growth rates and W-CIN

**Fig. 4:**
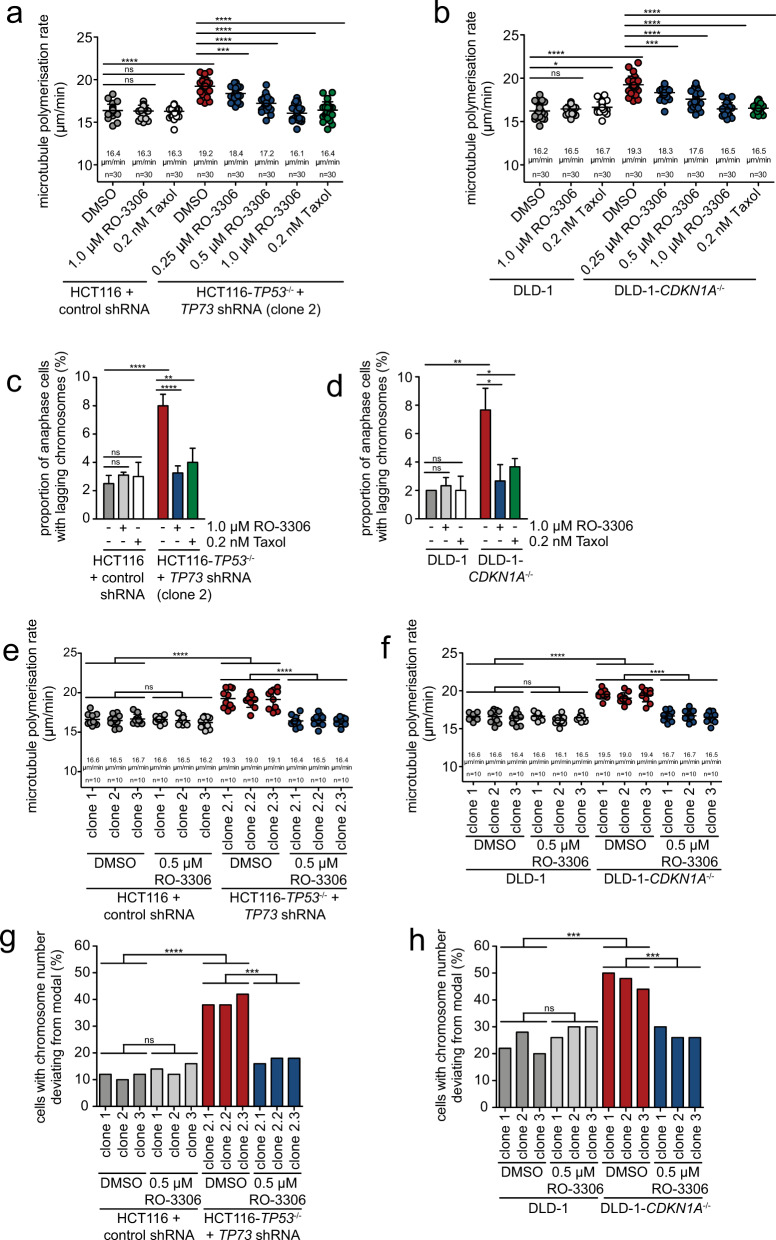
Inhibition of CDK1 suppresses abnormal microtubule polymerization rates and chromosomal instability after loss of p53/p73 or p21^CIP1^. **a** Rescue of abnormal microtubule growth rates upon partial CDK1 inhibition in *TP53/TP73*-deficient cells. HCT116 and *TP53/TP73*-deficient cells were treated with the CDK1 inhibitor RO-3306 or with low-dose Taxol for 16 h and mitotic microtubule plus end assembly rates were measured. Scatter dot plots show average microtubule polymerization rates (20 microtubules/cell, *n* = 30 mitotic cells from three independent experiments, mean ± SD, *t*-test). **b** Rescue of abnormal microtubule growth rates upon partial CDK1 inhibition in *CDKN1A*-deficient cells. DLD-1 and DLD-1-*CDKN1A*^−/−^ cells were treated as in **a** and mitotic microtubule polymerization rates were determined (20 microtubules/cell, *n* = 30 mitotic cells from three independent experiments, mean ± SD, *t*-test). **c** Suppression of lagging chromosomes upon partial CDK1 inhibition in *TP53/TP73*-deficient cells. HCT116 and *TP53/TP73*-deficient cells were treated with the CDK1 inhibitor RO-3306 or with low-dose Taxol. The graph shows the proportion of cells exhibiting lagging chromosomes during anaphase (*n* = 300 anaphase cells from three independent experiments, mean ± SD, *t*-test). **d** Suppression of lagging chromosomes upon partial CDK1 inhibition in *CDKN1A*-deficient cells. Parental and *CDKN1A*-deficient DLD-1 cells were treated as in **c**. The graph shows the proportion of cells exhibiting lagging chromosomes during anaphase (*n* = 300 anaphase cells from three independent experiments, mean ± SD, *t*-test). **e, f** Mitotic microtubule growth rates in single-cell clones derived from control, *TP53/TP73*-deficient HCT116 cells or from parental and *CDKN1A*-deficient DLD-1 cells and treated with or without CDK1 inhibitor (RO-3306). Scatter dot plots show average microtubule growth rates (20 microtubules/cell, *n* = 10 mitotic cells for each clone, mean ± SD, *t*-test). **g, h** Chromosome number variability in single-cell clones derived from control, *TP53/TP73*-deficient HCT116 cells or from parental and *CDKN1A*-deficient DLD-1 cells and treated with or without CDK1 inhibitor. The proportion of cells with a chromosome number deviating from modal (45 chromosomes for HCT116, 46 chromosomes for DLD-1) was calculated for three independent clones (*n* = 50 cells per condition).

p21^CIP1^ acts as a CDK inhibitor inhibiting CDK1 and CDK2 [24]. Since CDK1 is a key regulator of mitosis, we hypothesized that CDK1 activity might be unleashed upon loss of the p53/p73 - p21^CIP1^ axis and thereby trigger abnormal microtubule growth rates and chromosome missegregation in mitosis. To test this, we performed rescue experiments using the CDK1 specific inhibitor RO-3306 [37]. As expected, higher concentrations of RO-3306 resulted in cell cycle arrest in G2 before cells enter mitosis (Fig. S5a). We titrated very low doses of RO-3306 on control cells, *TP53/TP73*-deficient and *CDKN1A* knockout cells and found efficient restoration of proper microtubule growth rates and a suppression of lagging chromosomes (Fig. 4a–d, Fig. S5b). Remarkably, already very low doses of RO-3306 that did not affect cell cycle progression (Fig. S5a) were sufficient for these rescue effects suggesting that CDK1 activity is only mildly increased upon p53/p73 or p21^CIP1^ loss. To investigate an involvement of mildly increased CDK1 activity in the induction of W-CIN, we generated single-cell clones derived from control, *TP53/TP73*, or *CDKN1A*-deficient cells and grew them for 30 generations in the continuous absence or presence of the CDK1 inhibitor and determined the resulting chromosome number variability. In fact, weak inhibition of CDK1 was found to be sufficient to restore proper microtubule growth rates in the single-cell clones (Fig. 4e, f) and to suppress W-CIN in the absence of p53/p73 or p21^CIP1^ (Fig. 4g, h, Fig. S6a, b). It is of note that suppression of W-CIN and the evolvement of aneuploidy in these long-term experiments required even lower concentrations of RO-3306 (0.5 µM instead of 1.0 µM in the short-term experiments), which might be related to intra-cellular accumulation of the inhibitor upon prolonged treatment (Fig. 4a, b, Fig. S5b). Our results suggest that the inactivation of the p53/p73 - p21^CIP1^ axis mildly unleashes CDK1 activity and this causes abnormal microtubule growth rates and chromosome missegregation in mitosis.

### Mild unscheduled activation of CDK1 activity triggers increased microtubule growth rates and W-CIN

**Fig. 5:**
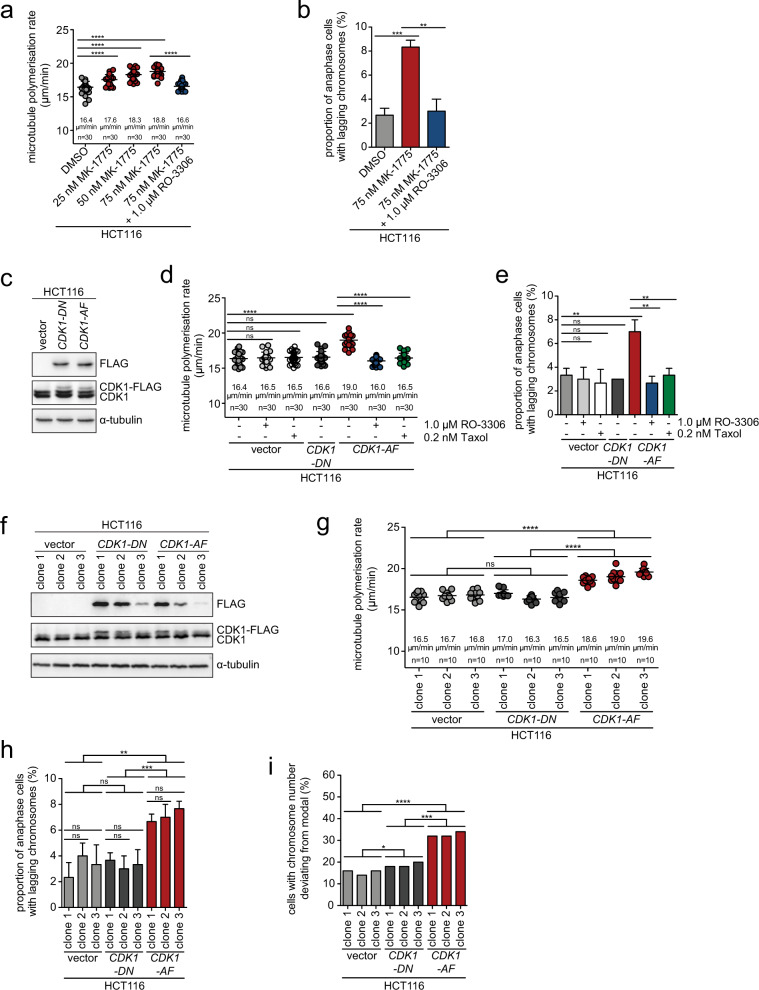
Increased CDK1 induces chromosomal instability. **a** Mitotic microtubule polymerization rates upon induction of CDK1 activity mediated by inhibition of wee1. HCT116 cells were treated with increasing concentrations of the wee1 inhibitor MK-1775 in the absence or presence of the CDK1 inhibitor RO-3306 and mitotic microtubule plus end assembly rates were determined. Average microtubule polymerization rates are depicted in scatter dot plots (20 microtubules/cell, *n* = 30 mitotic cells from three independent experiments, mean ± SD, t-test). **b** Induction of lagging chromosomes upon CDK1 activation mediated by wee1 inhibition. HCT116 cells were treated with MK-1775 in the absence or presence of RO-3306. The graph shows the proportion of anaphase cells exhibiting lagging chromosomes (*n* = 300 anaphase cells from three independent experiments, mean ± SD, *t*-test). **c** Transient expression of FLAG-tagged *CDK1-AF* (constitutive active) or *CDK1-DN* (inactive) in chromosomally stable HCT116 cells. A representative western blot detecting CDK1 protein levels and exogenously expressed CDK1 (FLAG) is shown. **d** Mitotic microtubule polymerization rates after transient expression of *CDK1-AF* or *CDK1-DN* in HCT116 cells. Average microtubule polymerization rates are depicted in scatter dot plots (20 microtubules/cell, *n* = 30 mitotic cells from three independent experiments, mean ± SD, *t*-test). **e** Quantification of cells exhibiting lagging chromosomes after transient expression of *CDK1-AF* or *CDK1-DN* in the absence or presence of RO-3306 or Taxol (*n* = 300 anaphase cells from three independent experiments, mean ± SD, *t*-test). **f** Generation of HCT116 cell lines stably expressing *CDK1-AF* or *CDK1-DN*. The expression level of the CDK1 variants was detected in three independent cell clones. A representative western blot is shown. **g** Mitotic microtubule polymerization rates in single-cell clones of HCT116 cells stably expressing *CDK1-AF* or *CDK1-DN*. Average microtubule polymerization rates are depicted in scatter dot plots (20 microtubules/cell, *n* = 10 mitotic cells for each independent single-cell clone, mean ± SD, *t*-test). **h** Quantification of cells exhibiting lagging chromosomes in HCT116 single-cell clones stably expressing *CDK1-AF* or *CDK1-DN* (*n* = 300 anaphase cells for each independent single-cell clone, mean ± SD, *t*-test). **i** Chromosome number variability in single-cell clones derived from HCT116 cells stably expressing *CDK1-AF* or *CDK1-DN*. The proportion of cells with a chromosome number deviating from modal (45 chromosomes for HCT116 cells) was calculated for three independent clones (*n* = 50 cells per single-cell clone).

We considered that direct, mild activation of CDK1 independently of the p53/p73 - p21^CIP1^ axis might result in increased microtubule growth rates and chromosome missegregation in mitosis. To directly activate CDK1, we inhibited the wee1 kinase that is known to inhibit CDK1 activity by direct phosphorylation of CDK1 on tyrosine-15 [38]. Titration experiments using the established wee1 inhibitor MK-1775 [39] verified that CDK1 is indeed dephosphorylated and activated in an inhibitor concentration-dependent manner leading to premature entry into mitosis (Fig. S7). Importantly, even very low concentrations of the wee1 inhibitor that cause only very little dephosphorylation of CDK1 and no premature entry into mitosis (Fig. S7) were already sufficient to increase mitotic microtubule assembly rates and to induce lagging chromosomes (Fig. 5a, b). Moreover, these effects were rescued upon mild inhibition of CDK1 by low-dose RO-3306 demonstrating that wee1 inhibition acts on microtubule dynamics and lagging chromosome induction through mildly increased CDK1 activity (Fig. 5a, b). Further, we directly elevated CDK1 by expression of a non-phosphorylatable, constitutively active mutant of *CDK1* (*CDK1-AF*) and, as a control, of a kinase-dead mutant of *CDK1* (*CDK1-DN*, Fig. 5c*)* [40]. Only expression of *CDK1-AF* triggered an increase in microtubule growth rates in mitosis and induced lagging chromosomes (Fig. 5d, e). These effects of *CDK1-AF* expression were found to be dependent on its kinase activity since the mitotic defects were suppressed by CDK1 inhibition using low doses of RO-3306 (Fig 5d, e). Also, Taxol treatment corrected normal microtubule polymerization rates and suppressed the generation of lagging chromosomes (Fig. 5d, e). To prove whether increased CDK1 activity is sufficient to induce W-CIN, we generated single-cell clones stably expressing either active *CDK1-AF* or kinase-dead *CDK1-DN* (Fig. 5f). Even very low-level expression of *CDK1-AF* was sufficient to increase microtubule growth rates (Fig. 5g), induced lagging chromosomes (Fig. 5h) and W-CIN (Fig. 5i, Fig. S8). Together, these results suggest that even mildly increased CDK1 activity as a result of the loss of the p53/p73 - p21^CIP1^ axis or upon inhibition of wee1 can act as a trigger for W-CIN by increasing microtubule assembly rates in mitosis.

### Mild inhibition of CDK1 is sufficient to suppress increased microtubule polymerization rates and chromosome missegregation in cancer cells

**Fig. 6:**
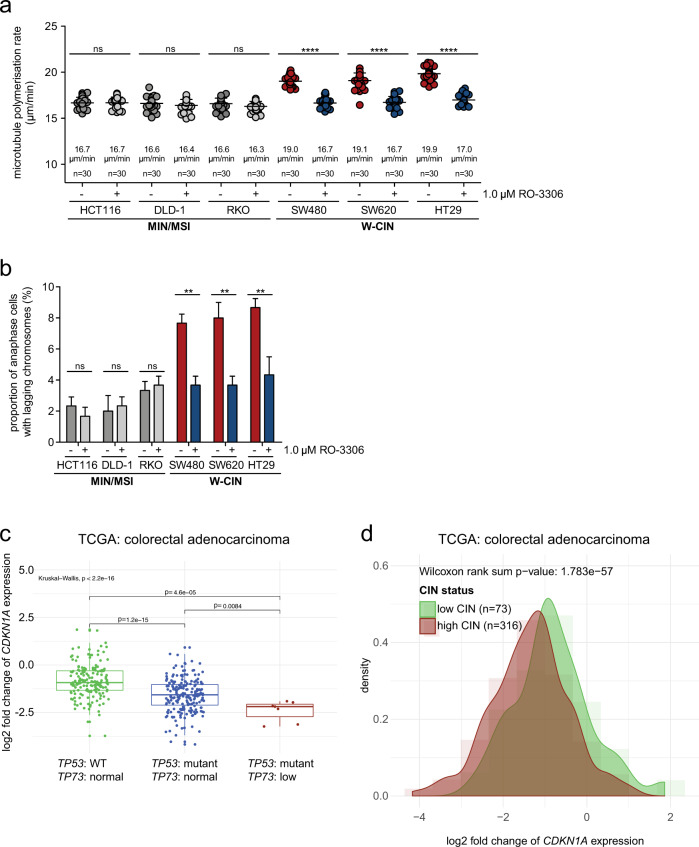
Relevance of the *TP53*, *TP73*, *CDKN1A*, and *CDK1* status for chromosomal instability in colorectal cancer. **a** Rescue of increased mitotic microtubule polymerization rates in various colorectal cancer cell lines exhibiting W-CIN by mild inhibition of CDK1. The indicated cell lines exhibiting either MIN/MSI or W-CIN were used to measure mitotic microtubule polymerization rates in the absence or presence of low concentrations of the CDK1 inhibitor RO-3306 (20 microtubules/cell, *n* = 30 mitotic cells from three independent experiments, mean ± SD, *t*-test). **b** Suppression of lagging chromosomes upon partial CDK1 inhibition in colorectal cancer cell lines exhibiting W-CIN. The indicated cell lines were treated with or without low concentrations of RO-3306 and the proportion of cells showing lagging chromosomes in anaphase was determined (*n* = 300 anaphase cells from three independent experiments, mean ± SD, *t*-test). **c** Relationship between *TP53* and *TP73* status to *CDKN1A* expression levels in colorectal cancer. 389 human tumor samples from The Cancer Genome Atlas (TCGA) were analyzed for their *TP53* mutational status and *TP73* expression level status, and its association with *CDKN1A* expression. The pair-wise *p* values were obtained from a Wilcoxon rank sum test. **d** The relationship between *CDKN1A* expression and chromosomal instability (weighted Genome Integrity Index, wGII). 389 colorectal adenocarcinoma samples from The Cancer Genome Atlas (TCGA) were categorized into low and high CIN groups and aligned to their expression level of *CDKN1A*. The combined density plot and histogram of *CDKN1A* gene expression indicates the shift of the distribution between high and low CIN scores.

**Fig. 7:**
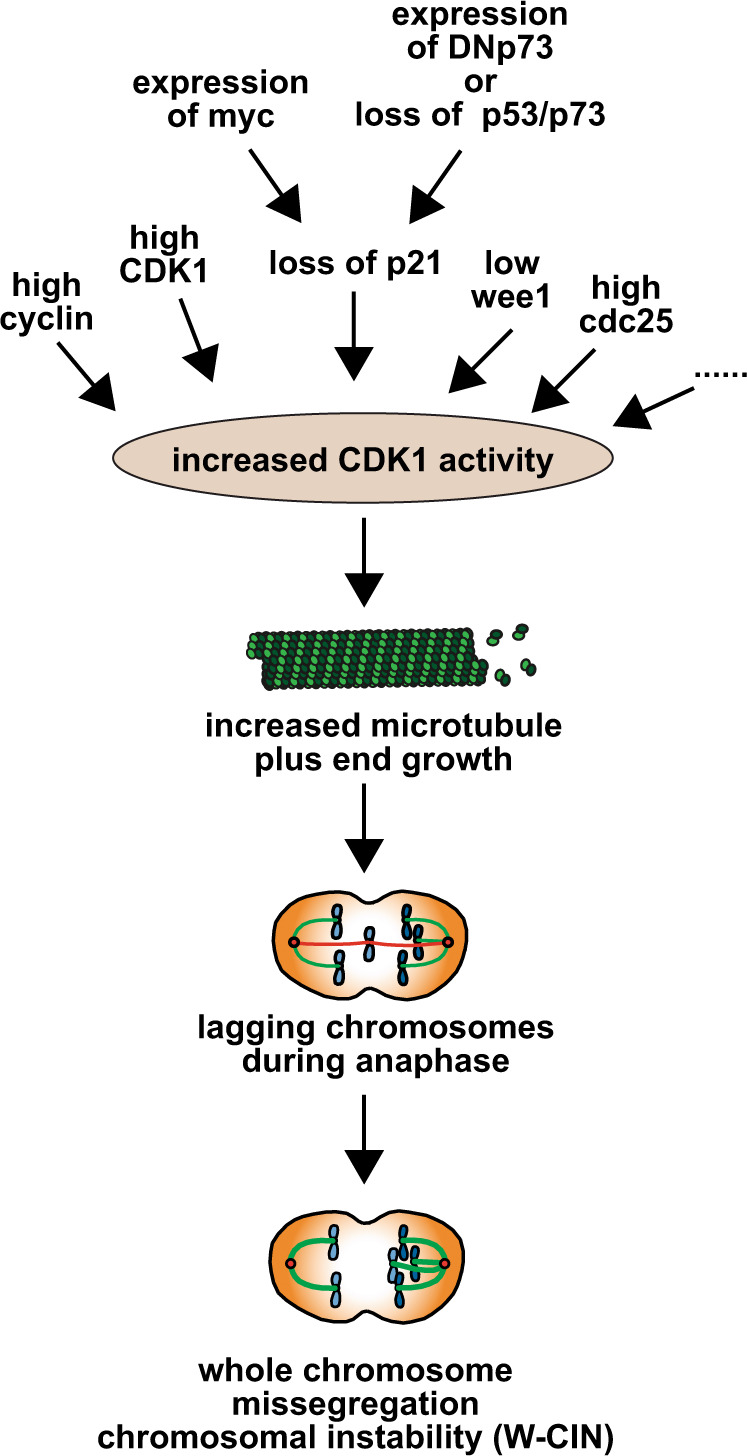
Model: increased CDK1 activity as a trigger for whole chromosome instability in human cancer. Several possible ways that lead to an increase in CDK1 activity in human cancer cells are illustrated. Examples are loss of the p53/p73 - p21^CIP1^ axis, loss of wee1 or overexpression of CDK1 or associated cyclins. In turn, CDK1 activity increases microtubule polymerization rates in mitosis leading to the generation of lagging chromosomes and causing whole chromosome missegregation and aneuploidy.

To test whether unleashed CDK1 activity can act as a trigger for increased microtubule polymerization rates and subsequent chromosome missegregation in human cancer cells we analyzed a panel of colorectal cancer (CRC) cell lines characterized by either MIN/MSI (chromosomally stable, nearly diploid) or W-CIN (chromosomally unstable, aneuploid). As shown previously [10], only CRC cell lines characterized by W-CIN exhibit increased microtubule growth rates and lagging chromosomes (Fig. 6a, b). We treated the different cell lines with low-doses of the CDK1 inhibitor RO-3306, and measured mitotic microtubule polymerization rates. In fact, mild CDK1 inhibition was sufficient to restore proper microtubule assembly rates in cell lines exhibiting W-CIN and had no effect in MIN/MSI cells (Fig. 6a). Consequently, lagging chromosomes and thus, chromosome missegregation were also significantly suppressed in W-CIN cell lines upon mild CDK1 inhibition (Fig. 6b). It is of note that we were not able to consistently associate alterations in p53, p73, p21^CIP1^ or CDK1 protein levels with increased microtubule polymerization rates in the different W-CIN cell lines (Fig. S9) suggesting that various mechanisms and alterations might contribute to CDK1 deregulation in human cancer cells (see model in Fig. 7).

### Alterations of *TP53/TP73* and *CDKN1A* are associated with W-CIN in human cancer

Our experimental data presented here indicate that loss of *TP53* and *TP73* can trigger W-CIN by mediating a loss of *CDKN1A*. This prompted us to investigate whether this situation is reflected in human cancer samples. We employed bioinformatics correlation analyses of 389 colorectal cancer (CRC) samples and 972 breast carcinoma samples and were able to detect cases with normal p53 status, with loss of p53, and concomitant loss of p53 and p73, the latter at rather low frequency. Importantly, we found a significant correlation of low *CDKN1A* expression with loss of *TP53* and *TP73* for CRC and breast cancer (Fig. 6c, Fig. S10a). We then categorized the tumor samples based on the calculated weighted genome integrity index (wGII) into low and high CIN groups and correlated the status of CIN with the expression level of *CDKN1A*. Interestingly, there was a significant correlation between low *CDKN1A* expression and the presence of chromosomal instability (Fig. 6d, Fig. S10b). Thus, these bioinformatic analyses support the hypothesis that loss of *CDKN1A* expression associated with unleashed CDK1 can be linked to CIN in human cancer. Given the observation that concomitant loss of *TP53* and *TP73* is rather infrequent in cancer (at least in CRC and breast cancer) this also suggests that routes other than p53/p73 loss can result in lowered expression of *CDKN1A* and increased CDK1 activity and contribute to abnormally increased microtubule polymerization rates and CIN in human cancer (Fig. 7).

## Discussion

Our work demonstrates that mildly increased CDK1 activity is sufficient to trigger whole chromosome missegregation in mitosis and thus, whole chromosome instability (W-CIN) in otherwise chromosomally stable human cells. In accordance with our previous work [10–12, 16], W-CIN in response to unleashed CDK1 is mediated by an abnormal increase in microtubule plus end polymerization rates within mitotic spindles. This particular mitotic defect is a characteristic of chromosomally unstable cancer cells and is defined by an approx. 20-30% increase in microtubule plus end polymerization rates without grossly affecting other microtubule dynamics parameters and without affecting the overall spindle size and structure [10]. It is currently not understood in detail how increased microtubule polymerization rates cause chromosome missegregation, but existing data indicate that abnormal microtubule polymerization rates trigger transient spindle mispositioning, thereby facilitating the generation of erroneous merotelic microtubule-kinetochore attachments and inducing lagging chromosomes during anaphase as a pre-stage of chromosome missegregation [10]. Hence, transient spindle positioning alterations might act similarly to transient multipolar spindle intermediates upon centrosome amplification or abnormal spindle geometry to increase the probability for the generation of erroneous merotelic microtubule-kinetochore attachments as the basis for the formation of lagging chromosomes [6, 7, 41, 42].

In our work presented here, we focused on the upstream events responsible for triggering abnormal microtubule polymerization in cancer cells. Specifically, we asked which cancer-relevant alterations can contribute to W-CIN in cancer cells. By screening for known oncogenes and tumor suppressor genes, we found that loss of the tumor suppressor gene *TP53* is not sufficient to trigger W-CIN, which is in line with former results [10, 18]. On the other hand, it is well known that loss of *TP53* is among the most significant cancer alterations associated with chromosomal instability [17]. It was suggested that the association between loss of *TP53* and CIN might be explained by the fact that loss of *TP53* creates a permissive situation for the proliferation of aneuploid cells and is therefore required for long-term proliferation of chromosomally unstable cancer cells [43]. However, this view has been challenged by the observation that chromosome missegregation at a level comparable to cancer cells does not obligatory result in a strong p53 response leading to cell cycle arrest [44]. Also, it has been well documented that chromosomally unstable, but p53 proficient cells are still able to proliferate. This applies for instance to *MAD2* or *CHK2* deficient HCT116 cells that are chromosomally unstable, but harbor functional p53 [45, 46]. Nevertheless, loss of p53 might still be crucial to improve the proliferation of aneuploid cancer cells, which are otherwise strongly growth impaired [10, 47, 48].

Our work now revealed that loss of p53 can contribute to the induction of W-CIN, but only if its cousin p73 is also impaired indicating that p53 and p73 collaborate to prevent aneuploidy. Indeed, concomitant loss of p53 and p73 or expression of *DNp73*, which is known to act as a trans-repressor of p53- and p73-dependent transcription [49], results in increased microtubule polymerization rates, chromosome missegregation, and W-CIN.

In contrast to *TP53*, *TP73* is rarely mutated in cancer, but loss of its expression can be detected in various human cancers. This might be related to promotor methylation or a loss of the genomic locus of *TP73* on chromosome 1p.36, which is frequently deleted e.g., in neuroblastomas [49, 50]. We were able to detect concomitant loss of p53 and p73 in colorectal and breast cancer samples, albeit at low frequency. However, a loss of the common p53/p73 downstream target *CDKN1A* seems to be more frequent in human cancer [51]. Along this line, it was shown that 50% of CRC cases show a loss of *CDKN1A* expression and this correlated with CIN, but not with MIN/MSI [52], which is in agreement with our results from colorectal and breast cancer showing that loss of *CDKN1A* correlates with the CIN status of the cancer samples. However, reduced levels of *CDKN1A* are not only a consequence of loss of p53/p73. Also increased promotor repression, which can be mediated e.g., by high levels of the myc oncogene [53], can account for reduced *CDKN1A* expression. Hence, different cancer-relevant alterations might contribute to downregulation of *CDKN1A* and thereby, possibly to CIN.

Our data implicate increased CDK1 activity as a mediator of CIN. This low level of additional CDK1 activity is not sufficient to induce premature entry into mitosis suggesting that a severe deregulation of cell cycle timing is unlikely to account for the observed mitotic defects. Instead, it seems more plausible that even minor changes in CDK1-mediated phosphorylation on specific target proteins can contribute to increased microtubule polymerization. However, these targets are not yet known. One example of such a candidate protein might be CLASP2, which was shown to be phosphorylated by CDK1 in mitosis and this might be involved in stabilization of microtubule-kinetochore attachments [54]. Interestingly, hyper-stable microtubule-kinetochore attachments, which might result from hyper-phosphorylation of CLASP2, have been associated with the induction of lagging chromosomes and whole chromosome missegregation in mitosis [9].

Our previous work has identified the tumor suppressors BRCA1 and CHK2 and the oncogenic kinase AURKA as regulators of mitotic microtubule growth rates and for W-CIN [10, 11, 13]. Whether the BRCA1-CHK2-AURKA axis is influenced by increased CDK1 activity needs to be further investigated.

Given the fact that unleashed CDK1 is central to the induction of CIN, one has to consider various routes that can lead to increased CDK1 activity in cancer cells (Fig. 7). As demonstrated here, functional inactivation of p53/p73 or loss of p21^CIP1^ can drive CIN in a CDK1-dependent manner. Direct overexpression of *CDK1* or of its activating subunit cyclin B (*CNNB1/2*) are obvious additional possibilities to achieve higher CDK1 activity in cancer. In fact, overexpression of *CDK1* or of *CNNB1/2* is frequently found in various human cancers and correlates with poor prognosis [41, 55]. Moreover, *CDK1* and *CNNB1/2* have been identified as CIN signature genes whose overexpression correlates with CIN in cancer [56]. In this regard it is also interesting to note that overexpression of *CNNB2*, which could also promote CDK1, was shown to result in spindle positioning defects, chromosome missegregation, and the induction of aneuploidy [41].

In addition to direct alterations in CDK1-cyclin B itself, also upstream regulators of CDK1 can potentially be altered in cancer cells and can be involved in the induction of CIN. In fact, a network of kinases and phosphatases are involved in the precise regulation of CDK1 activity at the G2/M transition (for review see: [57]). Among these upstream regulators, cdc25 phosphatases are known to act as activators of CDK activity and are frequently overexpressed in cancer [58]. Also, changes in the subcellular localization or a spectrum of mutations might account for functional alterations in upstream pathways leading to hyper-activation of CDK1.

It is intriguing that W-CIN associated with whole chromosome missegregation is not only leading to whole chromosome missegregation and aneuploidy, but also to the induction of structural chromosome aberrations. In fact, recent work has demonstrated that missegregation of a single chromosome can lead to its encapsulation into a micronucleus, which, in turn, is prone for chromotripsis leading to complex genomic rearrangements [59, 60]. Thus, W-CIN is considered a driver for the rapid development of genome alterations on both, a numerical and structural level and increased CDK1 activity might contribute to these chromosomal aberrations.

## Supplementary information

Supplementary Information

Supplementary Figure Legends

Supplemental Figure S1

Supplemental Figure S2

Supplemental Figure S3

Supplemental Figure S4

Supplemental Figure S5

Supplemental Figure S6

Supplemental Figure S7

Supplemental Figure S8

Supplemental Figure S9

Supplemental Figure S10

## References

[1] Bakhoum SF, Landau DA (2017). Chromosomal instability as a driver of tumor heterogeneity and evolution. Cold Spring Harb Perspect Med.

[2] Turajlic S, Sottoriva A, Graham T, Swanton C (2019). Resolving genetic heterogeneity in cancer. Nat Rev Genet.

[3] Sansregret L, Swanton C (2017). The role of aneuploidy in cancer evolution. Cold Spring Harb Perspect Med.

[4] Lengauer C, Kinzler KW, Vogelstein B (1998). Genetic instabilities in human cancers. Nature.

[5] Gregan J, Polakova S, Zhang L, Tolic-Norrelykke IM, Cimini D (2011). Merotelic kinetochore attachment: causes and effects. Trends Cell Biol.

[6] Ganem NJ, Godinho SA, Pellman D (2009). A mechanism linking extra centrosomes to chromosomal instability. Nature.

[7] Silkworth WT, Nardi IK, Scholl LM, Cimini D (2009). Multipolar spindle pole coalescence is a major source of kinetochore mis-attachment and chromosome mis-segregation in cancer cells. PloS One.

[8] Bakhoum SF, Thompson SL, Manning AL, Compton DA (2009). Genome stability is ensured by temporal control of kinetochore-microtubule dynamics. Nat Cell Biol.

[9] Bakhoum SF, Genovese G, Compton DA (2009). Deviant kinetochore microtubule dynamics underlie chromosomal instability. Curr Biol.

[10] Ertych N, Stolz A, Stenzinger A, Weichert W, Kaulfuss S, Burfeind P (2014). Increased microtubule assembly rates influence chromosomal instability in colorectal cancer cells. Nat Cell Biol.

[11] Ertych N, Stolz A, Valerius O, Braus GH, Bastians H (2016). CHK2-BRCA1 tumor-suppressor axis restrains oncogenic Aurora-A kinase to ensure proper mitotic microtubule assembly. Proc Natl Acad Sci USA.

[12] Luddecke S, Ertych N, Stenzinger A, Weichert W, Beissbarth T, Dyczkowski J (2016). The putative oncogene CEP72 inhibits the mitotic function of BRCA1 and induces chromosomal instability. Oncogene.

[13] Stolz A, Ertych N, Bastians H (2010). Loss of the tumour-suppressor genes CHK2 and BRCA1 results in chromosomal instability. Biochem Soc Trans.

[14] Stolz A, Neufeld K, Ertych N, Bastians H (2015). Wnt-mediated protein stabilization ensures proper mitotic microtubule assembly and chromosome segregation. EMBO Rep.

[15] Bohly N, Kistner M, Bastians H (2019). Mild replication stress causes aneuploidy by deregulating microtubule dynamics in mitosis. Cell Cycle.

[16] Stolz A, Ertych N, Bastians H (2015). Microtubule plus tips: a dynamic route to chromosomal instability. Mol Cell Oncol.

[17] Cancer Genome Atlas N. Comprehensive molecular characterization of human colon and rectal cancer. Nature. 2012;487:330–7. 10.1038/nature11252 PMC3401966 22810696

[18] Bunz F, Fauth C, Speicher MR, Dutriaux A, Sedivy JM, Kinzler KW (2002). Targeted inactivation of p53 in human cells does not result in aneuploidy. Cancer Res.

[19] Maas AM, Bretz AC, Mack E, Stiewe T (2013). Targeting p73 in cancer. Cancer Lett.

[20] Flores ER, Sengupta S, Miller JB, Newman JJ, Bronson R, Crowley D (2005). Tumor predisposition in mice mutant for p63 and p73: evidence for broader tumor suppressor functions for the p53 family. Cancer Cell.

[21] Engelmann D, Meier C, Alla V, Putzer BM (2015). A balancing act: orchestrating amino-truncated and full-length p73 variants as decisive factors in cancer progression. Oncogene.

[22] Di C, Yang L, Zhang H, Ma X, Zhang X, Sun C (2013). Mechanisms, function and clinical applications of DNp73. Cell Cycle.

[23] Warfel NA, El-Deiry WS (2013). p21WAF1 and tumourigenesis: 20 years after. Curr Opin Oncol.

[24] Medema RH, Klompmaker R, Smits VA, Rijksen G (1998). p21waf1 can block cells at two points in the cell cycle, but does not interfere with processive DNA-replication or stress-activated kinases. Oncogene.

[25] Kreis NN, Sanhaji M, Rieger MA, Louwen F, Yuan J (2014). p21Waf1/Cip1 deficiency causes multiple mitotic defects in tumor cells. Oncogene.

[26] Kreis NN, Louwen F, Yuan J (2019). The multifaceted p21 (Cip1/Waf1/CDKN1A) in cell differentiation, migration and cancer therapy. Cancers.

[27] Bunz F, Dutriaux A, Lengauer C, Waldman T, Zhou S, Brown JP (1998). Requirement for p53 and p21 to sustain G2 arrest after DNA damage. Science.

[28] Waldman T, Kinzler KW, Vogelstein B (1995). p21 is necessary for the p53-mediated G1 arrest in human cancer cells. Cancer Res.

[29] Schmidt M, Lu Y, Liu B, Fang M, Mendelsohn J, Fan Z (2000). Differential modulation of paclitaxel-mediated apoptosis by p21Waf1 and p27Kip1. Oncogene.

[30] Stepanova T, Slemmer J, Hoogenraad CC, Lansbergen G, Dortland B, De Zeeuw CI (2003). Visualization of microtubule growth in cultured neurons via the use of EB3-GFP (end-binding protein 3-green fluorescent protein). J Neurosci.

[31] Law CW, Chen Y, Shi W, Smyth GK (2014). voom: precision weights unlock linear model analysis tools for RNA-seq read counts. Genome Biol.

[32] Ritchie ME, Phipson B, Wu D, Hu Y, Law CW, Shi W (2015). limma powers differential expression analyses for RNA-sequencing and microarray studies. Nucleic Acids Res.

[33] Burrell RA, McClelland SE, Endesfelder D, Groth P, Weller MC, Shaikh N (2013). Replication stress links structural and numerical cancer chromosomal instability. Nature.

[34] Harms K, Nozell S, Chen X (2004). The common and distinct target genes of the p53 family transcription factors. Cell Mol Life Sci.

[35] Bates S, Ryan KM, Phillips AC, Vousden KH (1998). Cell cycle arrest and DNA endoreduplication following p21Waf1/Cip1 expression. Oncogene.

[36] Niculescu AB, Chen X, Smeets M, Hengst L, Prives C, Reed S (1998). Effects of p21^Cip1/Waf1^ at both the G_1_/S and the G_2_/M cell cycle transitions: pRb is a critical determinant in blocking DNA replication and in preventing endoreduplication. Mol Cell Biol.

[37] Vassilev LT, Tovar C, Chen S, Knezevic D, Zhao X, Sun H (2006). Selective small-molecule inhibitor reveals critical mitotic functions of human CDK1. Proc Natl Acad Sci USA.

[38] Schmidt M, Rohe A, Platzer C, Najjar A, Erdmann F, Sippl W (2017). Regulation of G2/M transition by inhibition of WEE1 and PKMYT1 kinases. Molecules.

[39] Hirai H, Iwasawa Y, Okada M, Arai T, Nishibata T, Kobayashi M (2009). Small-molecule inhibition of Wee1 kinase by MK-1775 selectively sensitizes p53-deficient tumor cells to DNA-damaging agents. Mol Cancer Ther.

[40] Krek W, Nigg EA (1991). Mutations of p34cdc2 phosphorylation sites induce premature mitotic events in HeLa cells: evidence for a double block to p34cdc2 kinase activation in vertebrates. EMBO J.

[41] Nam HJ, van Deursen JM (2014). Cyclin B2 and p53 control proper timing of centrosome separation. Nat Cell Biol.

[42] Silkworth WT, Cimini D (2012). Transient defects of mitotic spindle geometry and chromosome segregation errors. Cell Div.

[43] Thompson SL, Compton DA (2010). Proliferation of aneuploid human cells is limited by a p53-dependent mechanism. J Cell Biol.

[44] Santaguida S, Richardson A, Iyer DR, M’Saad O, Zasadil L, Knouse KA (2017). Chromosome Mis-segregation generates cell-cycle-arrested cells with complex karyotypes that are eliminated by the immune system. Dev Cell.

[45] Stolz A, Ertych N, Kienitz A, Vogel C, Schneider V, Fritz B (2010). The CHK2-BRCA1 tumour suppressor pathway ensures chromosomal stability in human somatic cells. Nat Cell Biol.

[46] Michel LS, Liberal V, Chatterjee A, Kirchwegger R, Pasche B, Gerald W (2001). MAD2 haplo-insufficiency causes premature anaphase and chromosome instability in mammalian cells. Nature.

[47] Williams BR, Prabhu VR, Hunter KE, Glazier CM, Whittaker CA, Housman DE (2008). Aneuploidy affects proliferation and spontaneous immortalization in mammalian cells. Science.

[48] Sheltzer JM, Amon A (2011). The aneuploidy paradox: costs and benefits of an incorrect karyotype. Trends Genet.

[49] Melino G, De Laurenzi V, Vousden KH (2002). p73: Friend or foe in tumorigenesis. Nat Rev Cancer.

[50] Dong S, Pang JC, Hu J, Zhou LF, Ng HK (2002). Transcriptional inactivation of TP73 expression in oligodendroglial tumors. Int J Cancer.

[51] Abbas T, Dutta A (2009). p21 in cancer: intricate networks and multiple activities. Nat Rev Cancer.

[52] Ogino S, Kawasaki T, Kirkner GJ, Ogawa A, Dorfman I, Loda M (2006). Down-regulation of p21 (CDKN1A/CIP1) is inversely associated with microsatellite instability and CpG island methylator phenotype (CIMP) in colorectal cancer. J Pathol.

[53] Garcia-Gutierrez L, Delgado MD, Leon J (2019). MYC oncogene contributions to release of cell cycle brakes. Genes.

[54] Maia ARR, Garcia Z, Kabeche L, Barisic M, Maffini S, Macedo-Ribeiro S (2012). Cdk1 and Plk1 mediate a CLASP2 phospho-switch that stabilizes kinetochore-microtubule attachments. J Cell Biol.

[55] Li J, Wang Y, Wang X, Yang Q (2020). CDK1 and CDC20 overexpression in patients with colorectal cancer are associated with poor prognosis: evidence from integrated bioinformatics analysis. World J Surg Oncol.

[56] Carter SL, Eklund AC, Kohane IS, Harris LN, Szallasi Z (2006). A signature of chromosomal instability inferred from gene expression profiles predicts clinical outcome in multiple human cancers. Nat Genet.

[57] Crncec A, Hochegger H (2019). Triggering mitosis. FEBS Lett.

[58] Sur S, Agrawal DK (2016). Phosphatases and kinases regulating CDC25 activity in the cell cycle: clinical implications of CDC25 overexpression and potential treatment strategies. Mol Cell Biochem.

[59] Zhang C, Spektor A, Cornils H, Francis JM, Jackson EK, Liu S (2015). Chromothripsis from DNA damage in micronuclei. Nature.

[60] Umbreit NT, Zhang C, Lynch LD, Blaine LJ, Cheng AM, Tourdot R (2020). Mechanisms generating cancer genome complexity from a single cell division error. Science.

