# Supplementary material for: The p53/p73 - p21CIP1 tumor suppressor axis guards against chromosomal instability by restraining CDK1 in human cancer cells
Source: Oncogene. 2020 Nov 9;40(2):436–51. doi: 10.1038/s41388-020-01524-4 (PMC7808936; doi:10.1038/s41388-020-01524-4)
Supplement: Supplementary file 1 — Supplementary Information [file 41388_2020_1524_MOESM1_ESM.docx]

**Supplementary Information**

**Supplementary Material and Methods**

- **Cell culture**
- DLD1-1, DLD-1-*CDKN1A*^-/-^, HCT116, HCT116-*CDKN1A*^-/-^, HCT116-*TP53*^-/-^, HT29, RKO, SW480, and SW620 cells were cultured in RPMI1640 (PAN-Biotech GmbH, Germany) supplemented with 10% fetal bovine serum (FBS; Corning, USA), 100 units/ml penicillin and 100 μg/ml streptomycin (PAN-Biotech GmbH, Germany). RKO-p21-Pon cells were grown in DMEM (PAN-Biotech GmbH, Germany) supplemented with 10% FBS, 100 units/ml penicillin, 100 μg/ml streptomycin, and 500 µg/ml G418 (Santa Cruz, USA). All cell lines were grown at 37°C and 5% CO_2_. All cell lines were tested for mycoplasma contamination.
- **Cell treatments**
- Cells were treated with 0.2 nM Taxol (Sigma-Aldrich, Germany) to restore proper microtubule plus end assembly rates as described previously ^10^. To inhibit CDK1 activity, cells were treated with 0.25 µM - 7.0 µM RO-3306 (Santa Cruz, USA). To increase CDK1 activity, the wee1 inhibitor MK-1775 (Selleck Chemicals, USA) was used (25 nM - 5.0 µM). As control, corresponding volumes of DMSO or H_2_O were used. To induce *CDKN1A* expression, inducible RKO-p21-Pon cells were incubated with 0.25 µM - 5.0 µM ponasterone A (Santa Cruz, USA). To induce *DNp73* expression, HCT116-DNp73 cells were treated with 200 ng/ml doxycycline (Sigma-Aldrich, Germany)

**Plasmid and siRNA transfections**

For microtubule plus end tracking experiments, cells were transfected with 10 µg pEGFP-EB3 (kindly provided by Linda Wordeman, Seattle, WA, USA) using the GenePulser Xcell (Bio-Rad Laboratories, USA) at the following settings: 500 µF and 300 V (for HCT116 and SW620 cells), 950 µF and 200 V (for RKO cells), 950 µF and 220 V (for DLD-1, SW480 and HT29 cells). RKO-p21-Pon cells were transfected with 0.75 µg pEGFP-EB3 plasmid using ScreenFect®A according to the manufacturer´s protocol (ScreenFect GmbH, Germany). Transient expression of pcDNA3.1-FLAG-*CDK1-DN/AF* plasmids (kindly provided by Makoto Iimori, Kyushu, Japan and Lienhard Schmitz, Giessen, Germany) ^61^ was achieved upon transfection of HCT116 with 1 µg plasmid using ScreenFect®A (ScreenFect GmbH, Germany) following the manufacturer’s instructions. siRNAs (Sigma-Aldrich, Germany) were transfected using ScreenFect®siRNA (ScreenFect GmbH, Germany) according to the manufacturer’s instructions. 48 hours after transfection, further experiments were performed and transfection efficiency was analysed by Western blotting. The following siRNA sequences were used:

- *LUCIFERASE (LUC):* 5’-CUUACGCUGAGUACUUCGAUU-3’;
- *CKAP5:* 5’-GAGCCCAGAGUGGUCCAAA-3’
  *TP53:* 5’-GACUCCAGUGGUAAUCUAC-3’
  *TP73:* 5’-CAGGUGACCGACGUCGUGAAA-3’, 5’-CUCGGGAGGGACUUCAACGAA-3’, 5’-CCCGGGAUGCUCAACAACCAU-3’, 5’-CCCGCUCUUGAAGAAACUCUA-3’

**Generation of stable cell lines**

To generate stable HCT116 cell lines expressing *DNp73α* in a doxycycline-inducible manner, cells were transfected with pInducer20- *DNp73α* (kindly provided by Thorsten Stiewe, Marburg, Germany) using METAFECTENE (Biontex, Germany) according to the manufacturer´s instructions. Single cell clones were selected in medium containing 300 µg/ml G418 (Santa Cruz, USA). Stable cell lines expressing *TP73* shRNA were generated by transfection with pLKO.1-sh-*TP73* (Sigma-Aldrich, Germany; targeting sequence: 5’-CTCTCCTTCCTGTGTGTCCAA-3’) and single cell clones were selected in medium containing 1 µg/ml puromycin (Sigma-Aldrich, Germany). HCT116 cell lines stably expressing *CDK1* (catalytic inactive (DN) or constitutive active (AF) mutants) were obtained by stable transfection using pcDNA3.1-FLAG-*CDK1-DN/AF* plasmids (kindly provided by Makoto Iimori, Kyushu, Japan and Lienhard Schmitz, Giessen, Germany) ^61^. Single cell clones were selected in medium containing 300 µg/ml G418 (Santa Cruz, USA).

- **FACS analyses**
- Cells were fixed with 70% ethanol at 4°C overnight. To determine the mitotic index of the cell population, mitotic phospho-epitopes were stained with an anti-phospho-Ser/Thr-Pro MPM-2 antibody (1:1600, Merck Millipore, Germany, cat no 05-368) and a secondary antibody conjugated to Alexa-Fluor488 (1:2000, Thermo Fisher, USA, cat no A11029) as described previously ^45^. To analyse the DNA content and thus the cell cycle phase, cells were stained with the DNA intercalating agent propidium iodide after treatment with 1 µg/ml RNaseA (Applichem, Germany). Cells were measured using a BD FACSCanto II flow cytometer (Becton Dickinson, USA) and data analysis was performed with the software BD FACSDiva (Becton Dickinson, USA).
- **Measurement of microtubule plus end assembly rates**

To determine microtubule plus end assembly rates, live-cell microscopy was performed on cells expressing GFP-tagged end-binding protein 3 (EB3), which allows the visualisation of microtubule plus-tips ^10, 30^. Cells were transfected with pEGFP-*EB3* plasmid and 48 hours after transfection, cells were treated with 2 µM Dimethylenastron (DME; Calbiochem, USA) for 1 hour to synchronize cells in prometaphase of mitosis as described ^10-12^. Live cells were recorded using a DeltaVision Elite microscope (GE Healthcare, UK) equipped with a PCO Edge sCMOS camera (PCO, Germany) and the softWoRx® 6.0 Software Suite (GE Healthcare, USA). Images were taken every 2 seconds for 30 seconds in total. During image acquisition, cells were incubated at 37°C and 5% CO_2_. Images were deconvolved and analysed using the softWoRx® 6.0 Software Suite (GE Healthcare, USA). Average microtubule plus end growth rates were calculated from 10-30 cells with 20 microtubules measured per cell.

- **Detection of lagging chromosomes**
- To accumulate cells in anaphase, cells were synchronised with a double thymidine block and released into fresh growth medium for 8.5-9 hours as described ^10, 12^. Cells were fixed with 2% PFA for 5 minutes at room temperature and then with 100% methanol for 5 minutes at -20°C. Subsequently, the samples were blocked with 5% FBS in PBS for 25 minutes. To detect the mitotic spindle, centromeres, and DNA, cells were stained with anti-α-tubulin (1:700, B-5-1-2, Santa Cruz, USA, cat no sc-23948), anti-CENP-C (1:1000, MBL International Corporation, USA, cat no PD030), secondary antibodies conjugated to Alexa-Fluor488 and Alexa-Fluor594 (1:1000, Thermo Fisher, USA, cat no A11029, A11076), and Hoechst33342 (1:15000, Thermo Fisher Scientific, USA). To determine the proportion of anaphase cells exhibiting lagging chromosomes, at least 300 anaphase cells were analysed from 3 independent experiments using a Leica DMI6000B fluorescence microscope (Leica, Germany) equipped with a Leica DFC360 FX camera (Leica, Germany) and the Leica LAS AF software (Leica, Germany). Only chromosomes, which were stained with both Hoechst33342 and anti-CENP-C and were clearly separated from the two DNA masses in anaphase, were defined as lagging.
- **Generation of single cell clones and W-CIN analysis**
- To analyse induction or suppression of W-CIN, single cell clones were grown for 30 cell cycles and the evolved chromosome number variability was determined on a single cell level. To analyse the effect of *DNp73* expression, doxycycline-inducible HCT116 cells were seeded at low density in the absence or presence of 200 ng/ml doxycycline (Sigma-Aldrich, Germany) and single cell clones were analysed after 30 days. To investigate the effect of restoration of proper microtubule growth rates, single cell clones were grown in the presence of 0.2 nM Taxol for 30 generations. Similarly, single cell clones were generated in the absence or presence of 0.5 µM RO-3306 (CDK1 inhibitor) or after expression of *CDK1-AF* or *CDK1-DN*. Mitotic chromosome spreads were obtained upon treatment with 2 µM Dimethylenastron (DME; Calbiochem, USA) for 4 hours to accumulate mitotic cells. Cells were harvested, incubated in hypotonic solution (40% RPMI1640 + 60% ddH_2_O) and fixed in ice-cold 75% methanol + 25% acetic acid. Subsequently, cells were resuspended in 100% acetic acid and carefully dropped onto pre-cooled glass slides as described previously ^10, 12^. After drying, cells were stained with Giemsa solution (Sigma-Aldrich, Germany). For each condition, the chromosome number of 50 metaphase spreads was counted using a Zeiss Axioscope FS microscope (Zeiss, Germany) equipped with a Hamamatsu digital camera C4742-95 (Hamamatsu Photonics, Japan) and the Hokawo Launcher 2.1 software (Hamamatsu Photonics, Japan).
- **Western blotting**
- Cells were lysed in lysis buffer (50 mM Tris-HCl, pH 7.4, 150 mM NaCl, 5 mM EDTA, 5 mM EGTA, 1% (v/v) NP-40, 0.1% (w/v) SDS, 0.1% (w/v) sodium deoxycholate, phosphatase inhibitor cocktail (25 mM β-glycerophosphate, 50 mM NaF, 5 mM Na_2_MoO_4_, 0.2 mM Na_3_VO_4_, 5 mM EDTA, 0.5 µM microcystin), protease inhibitor cocktail (Roche, Switzerland)). Proteins were separated on 7% or 11% SDS polyacrylamide gels and transferred to nitrocellulose membranes. The following antibodies were used in the indicated dilutions: anti-α-tubulin (1:1000, B-5-1-2, Santa Cruz, USA, cat no sc-23948), anti-β-actin (1:10000, AC-15, Sigma-Aldrich, Germany, cat no A5441), anti-CDK1 (1:500, Santa-Cruz, USA, cat no sc-54), anti-phospho-Y15-CDK1 (1:1000, Cell Signaling Technology, USA, cat no #9111), anti-ch-TOG (1:300, H-4, Santa Cruz, USA, cat no sc-374394), anti-FLAG (1:500, M2, Sigma-Aldrich, Germany, cat no F3165), anti-p21 (1:1000, DCS60, Cell Signaling Technology, USA, cat no #2946), anti-p53 (1:500, DO-1, Santa Cruz, USA, cat no sc-126), anti-p73 (1:1000, EP436Y, Abcam, UK, cat no ab40658), secondary antibodies conjugated to horseradish peroxidase (1:10000, Jackson ImmunoResearch, USA, cat no 115-035-146, 111-035-144). Proteins were detected by enhanced chemiluminescence.

**Supplementary references**

61. Seibert M, Kruger M, Watson NA, Sen O, Daum JR, Slotman JA *et al*. CDK1-mediated phosphorylation at H2B serine 6 is required for mitotic chromosome segregation. *J Cell Biol* 2019; **218**: 1164-1181.
