# Supplementary material for: The p53/p73 - p21CIP1 tumor suppressor axis guards against chromosomal instability by restraining CDK1 in human cancer cells
Source: Oncogene. 2020 Nov 9;40(2):436–51. doi: 10.1038/s41388-020-01524-4 (PMC7808936; doi:10.1038/s41388-020-01524-4)
Supplement: Supplementary file 2 — Supplementary Figure Legends [file 41388_2020_1524_MOESM2_ESM.docx]

**Supplemental Figure legends**

**Figure S1. Suppression of increased microtubule polymerization rates and of the generation of lagging chromosomes upon partial depletion of *CKAP5* (ch-TOG).**

**(A)** Partial siRNA mediated downregulation of *CKAP5* in control and *TP53/TP73*-deficient HCT116 cells. A representative western blot is shown. **(B)** Mitotic microtubule plus end assembly rates in parental HCT116 and HCT116 cells with loss of *TP53/TP73* after partial depletion of *CKAP5*. Scatter dot plots show average microtubule polymerization rates (20 microtubules/cell, n = 30 mitotic cells from 3 independent experiments, mean ­­± SD, *t*-test). **(C)** Proportion of cells exhibiting lagging chromosomes after loss of *TP53/TP73* and after partial depletion of *CKAP5* (n = 300 anaphase cells from 3 independent experiments, mean ± SD, *t*-test).

**Figure S2. Induction of chromosome number variability upon loss of *TP53* and *TP73*.**

Proportion of cells harbouring the indicated chromosome numbers per cell (n = 50 metaphase spreads for each single cell clone as indicated). This figure refers to Figure 2D.

**Figure S3. Induction of increased mitotic microtubule growth rates and chromosome number variability upon loss of *CDKN1A.***

**(A)** Loss of p21^CIP1^ protein levels in HCT116-*CDKN1A*^-/-^ cells. A representative western blot detecting p21^CIP1^ protein levels in HCT116 and HCT116-*CDKN1A*^-/-^ cells is shown. **(B)** Microtubule plus end assembly rates in mitotic HCT116 and HCT116-*CDKN1A*^-/-^ cells in the absence or presence of low-dose Taxol. Average microtubule polymerisation rates are shown in the scatter dot plots. (20 microtubules/cell, n = 30 mitotic cells from 3 independent experiments, mean ­­± SD, *t*-test). **(C)** Downregulation of p21^CIP^ protein levels after acute depletion of p53 and p73. HCT116 cells were treated with control or *TP53/TP73* siRNAs and protein levels for p53, p73 and p21^CIP1^ were detected by western blotting. A representative western blot is shown. **(D)** Microtubule plus end assembly rates after acute depletion of p53 and p73. HCT116 were treated with control or *TP53/TP73* siRNAs and average microtubule polymerisation rates were determined after 48 hours. Results are shown in the scatter dot plots. (20 microtubules/cell, n = 30 mitotic cells from 3 independent experiments, mean ­­± SD, *t*-test). **(E)** Mitotic microtubule polymerisation rates in single cell clones derived from DLD-1 and DLD-1-*CDKN1A*^-/-^ treated with either DMSO or 0.2 nM Taxol for 30 generations. Scatter dot plots show average microtubule polymerisation rates (20 microtubules/cell, n = 10 mitotic cells, mean ­­± SD, *t*-test). **(F)** Proportion of cells harbouring the indicated chromosome numbers per cell (n = 50 metaphase spreads for each single cell clones as indicated). This figure refers to Figure 3D.

**Figure S4. Induced expression of *CDKN1A* in chromosomally stable RKO cells.**

**(A)** Ponasterone-A-inducible expression of *CDKN1A* (encoding for p21^CIP1^) in RKO-p21-Pon cells. Cells were treated with increasing concentrations of ponasterone A for 24 hours and expression of *CDKN1A* was assessed. A representative western blot is shown. **(B)** High level expression of *CDKN1A* causes cell cycle arrest. RKO-p21-Pon cells were treated with increasing concentrations of ponasterone A for 24 hours to induce *CDKN1A* expression and FACS analysis was performed. Representative flow cytometry profiles are shown.

**Figure S5. Severe inhibition of CDK1 causes cell cycle arrest in G2 and *CDKN1A* deficient HCT116 cells exhibit inceased mitotic microtubule growth rates that are suppressed upon CDK1 inhbition.
(A)** Asynchronously growing control HCT116 and *TP53/TP73*-deficient HCT116 cells were incubated with increasing concentrations of RO-3306 for 16 hours. The DNA content was determined by FACS analyses. Representative flow cytometry profiles are shown. **(B)** Mitotic microtubule polymerisation rates in HCT116 and HCT116-*CDKN1A*^-/-^ cells in the absence or presence of 0.25 – 1.0 µM RO-3306 or 0.2 nM Taxol. Scatter dot plots show average microtubule polymerisation rates (20 microtubules/cell, n = 30 mitotic cells, mean ­­± SD, *t*-test).

**Figure S6. Induction of chromosome number variability upon loss of *TP53/TP73* or *CDKN1A* is suppressed by low-level CDK1 inhibition.
(A)** Proportion of control or *TP53/TP73* deficient HCT116 cells harbouring the indicated chromosome numbers per cell (n = 50 metaphase spreads for each single cell clone as indicated). This figure refers to Figure 4G. **(B)** Proportion of control or *CDKN1A* deficient DLD-1 cells harbouring the indicated chromosome numbers per cell (n = 50 metaphase spreads for each single cell clone as indicated). This figure refers to Figure 4H.

**Figure S7. Induction of CDK1 dephosphorylation and activation by wee1 inhibition.**

Western blot detecting phosphorylated (inactive) CDK1 in response to treatment with increasing concentrations of the wee1 inhibitor MK-1775 in HCT116 cells.

A representative western blot is shown.

**Figure S8. Induction of chromosome number variability upon increasing CDK1.**

Proportion of control or *CDK1-AF* or *CDK1-DN* expressing HCT116 cells harbouring the indicated chromosome numbers (n = 50 metaphase spreads for each single cell clone as indicated). This figure refers to Figure 5I.

**Figure S9. P53, p73, CDK1 and p21^CIP1^ protein levels in MIN/MSI and W-CIN cell lines.**

A representative western blot detecting p53, p73, CDK1 and p21^CIP1^ protein levels in the indicated chromosomally stable (MIN/MSI) or unstable (W-CIN) cell lines.

**Figure S10. Relevance of the *TP53*, *TP73* and *CDKN1A* status to chromosomal instability in breast cancer.**

**(A)** Relationship between *TP53* and *TP73* status to *CDKN1A* expression levels in breast carcinomas. 972 human tumour samples from The Cancer Genome Atlas (TCGA) were analysed for their *TP53* mutational status and *TP73* expression level status and its association with *CDKN1A* expression. Wilcoxon p-values were calculated for pair-wise comparisons and Kruskal-Wallis p-value for overall differences. **(B)** Relationship between *CDKN1A* expression and chromosomal instability (weighted Genome Integrity Index). 972 breast carcinoma samples from The Cancer Genome Atlas (TCGA) were categorized into low and high CIN groups and aligned to their expression level of *CDKN1A*. The combined histogram and density plot displays the distribution of *CDKN1A* expression in the low and high CIN groups.
