# Supplementary figures and images for: The p53/p73 - p21CIP1 tumor suppressor axis guards against chromosomal instability by restraining CDK1 in human cancer cells
Source: Oncogene. 2020 Nov 9;40(2):436–51. doi: 10.1038/s41388-020-01524-4 (PMC7808936; doi:10.1038/s41388-020-01524-4)

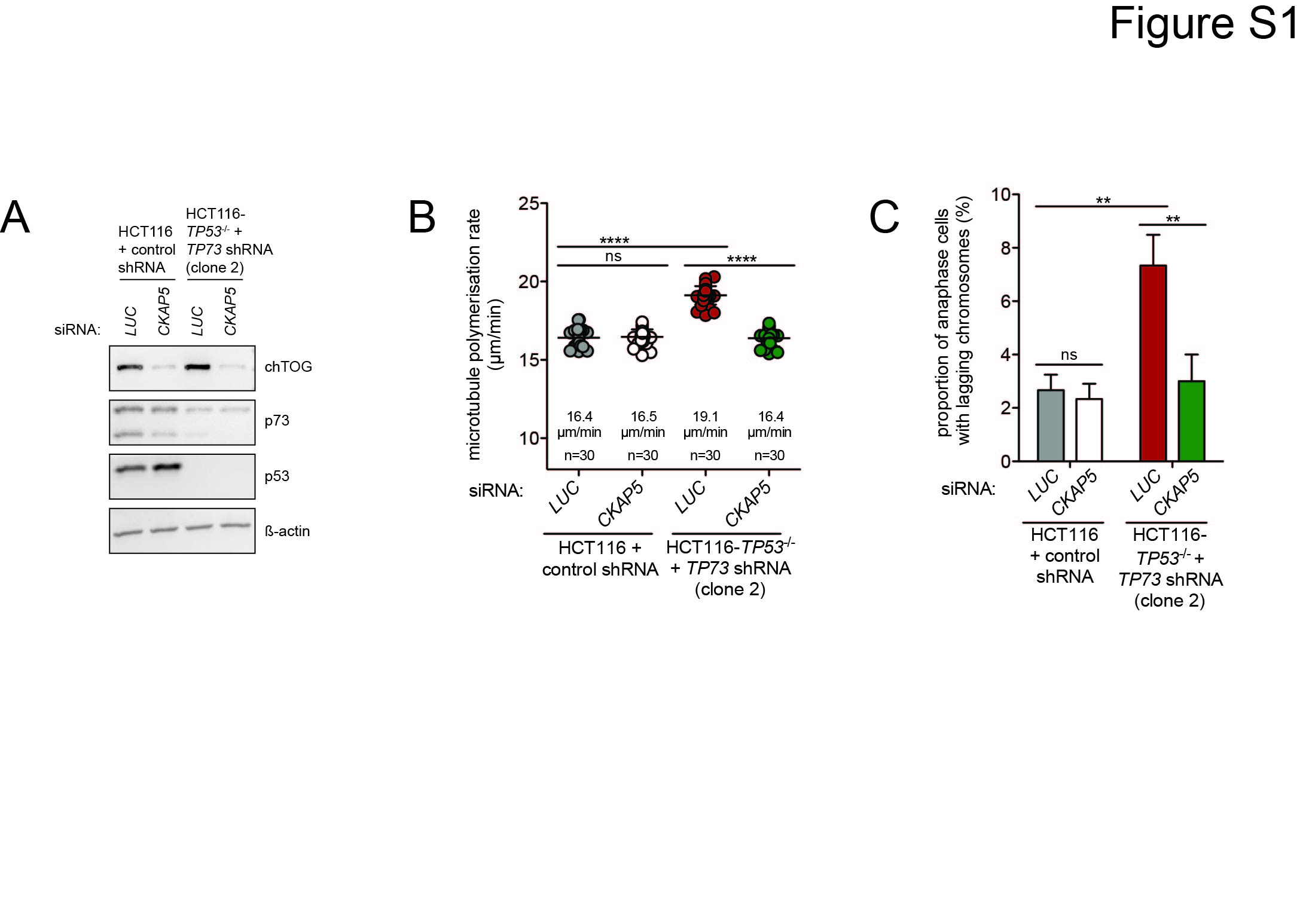

Supplement: Supplementary file 3 — Supplemental Figure S1 [file 41388_2020_1524_MOESM3_ESM.jpg]

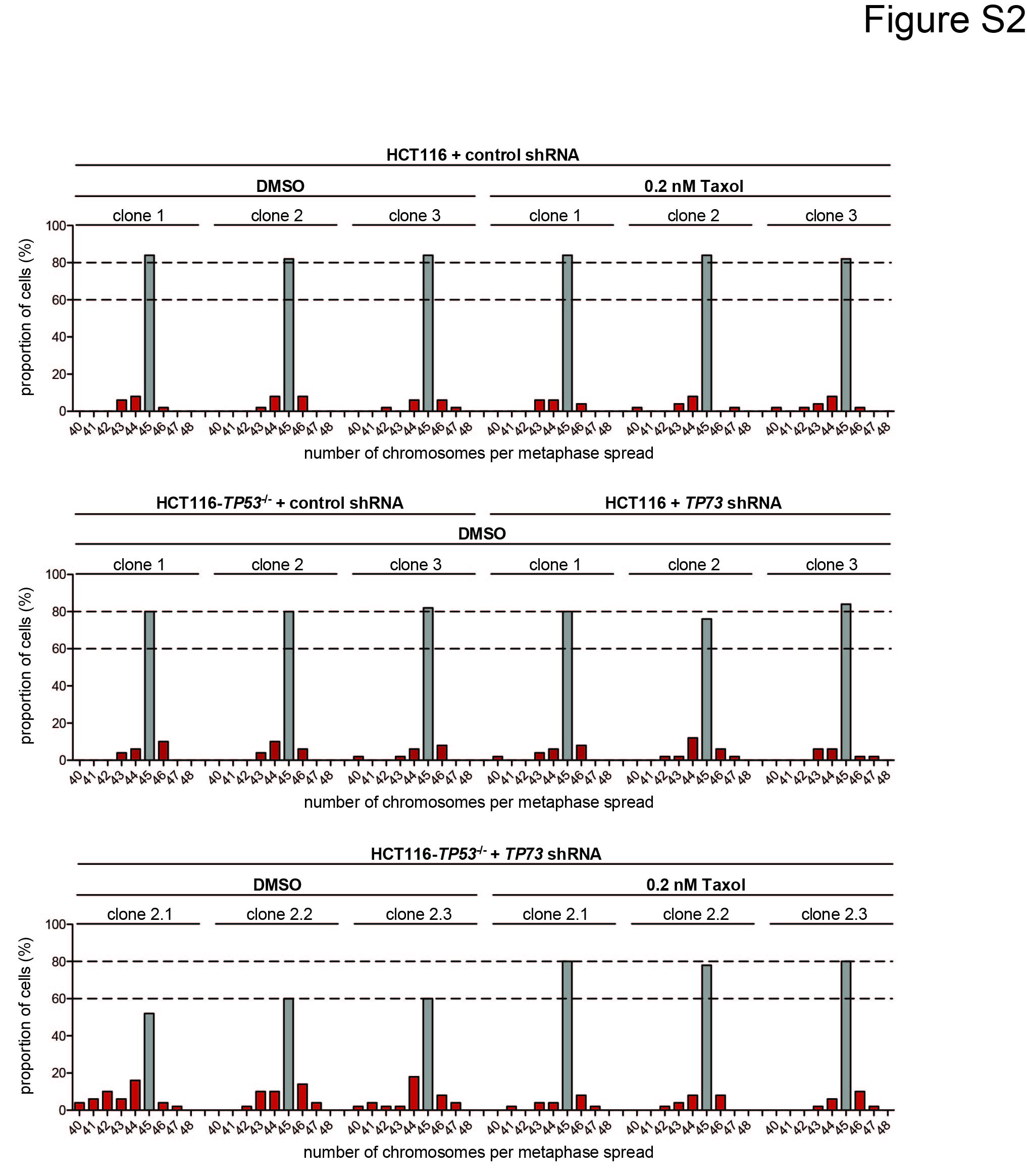

Supplement: Supplementary file 4 — Supplemental Figure S2 [file 41388_2020_1524_MOESM4_ESM.jpg]

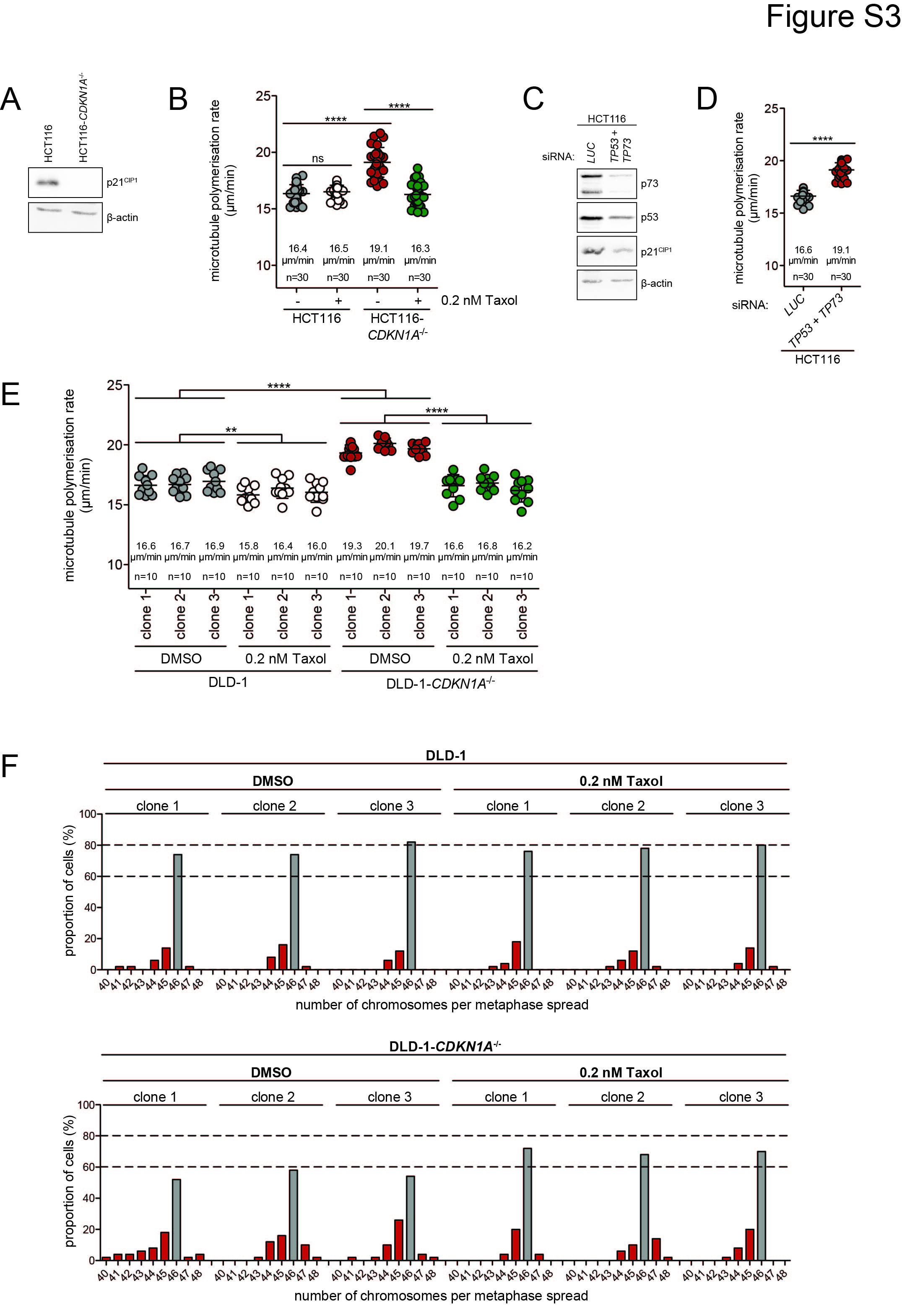

Supplement: Supplementary file 5 — Supplemental Figure S3 [file 41388_2020_1524_MOESM5_ESM.jpg]

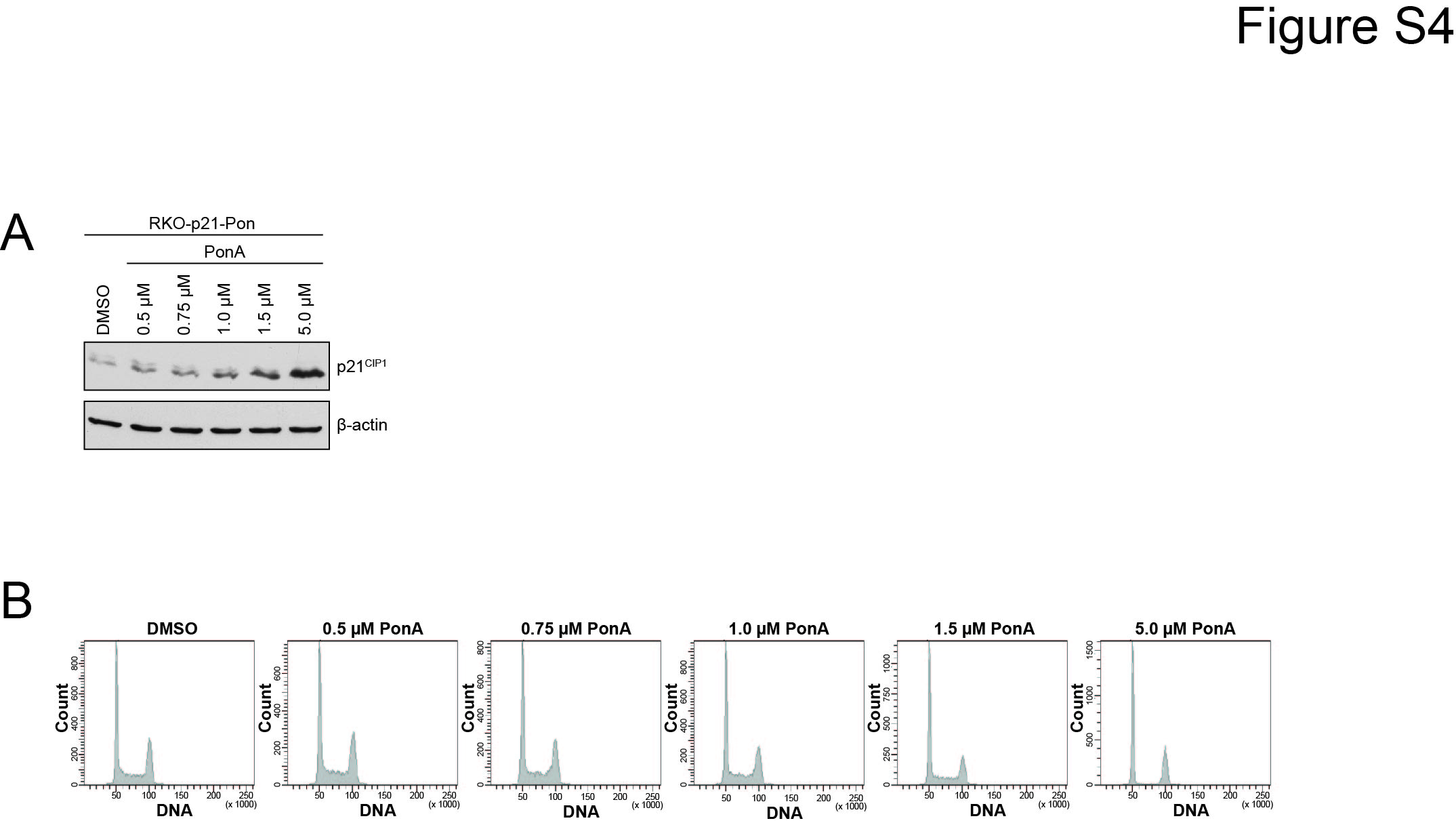

Supplement: Supplementary file 6 — Supplemental Figure S4 [file 41388_2020_1524_MOESM6_ESM.jpg]

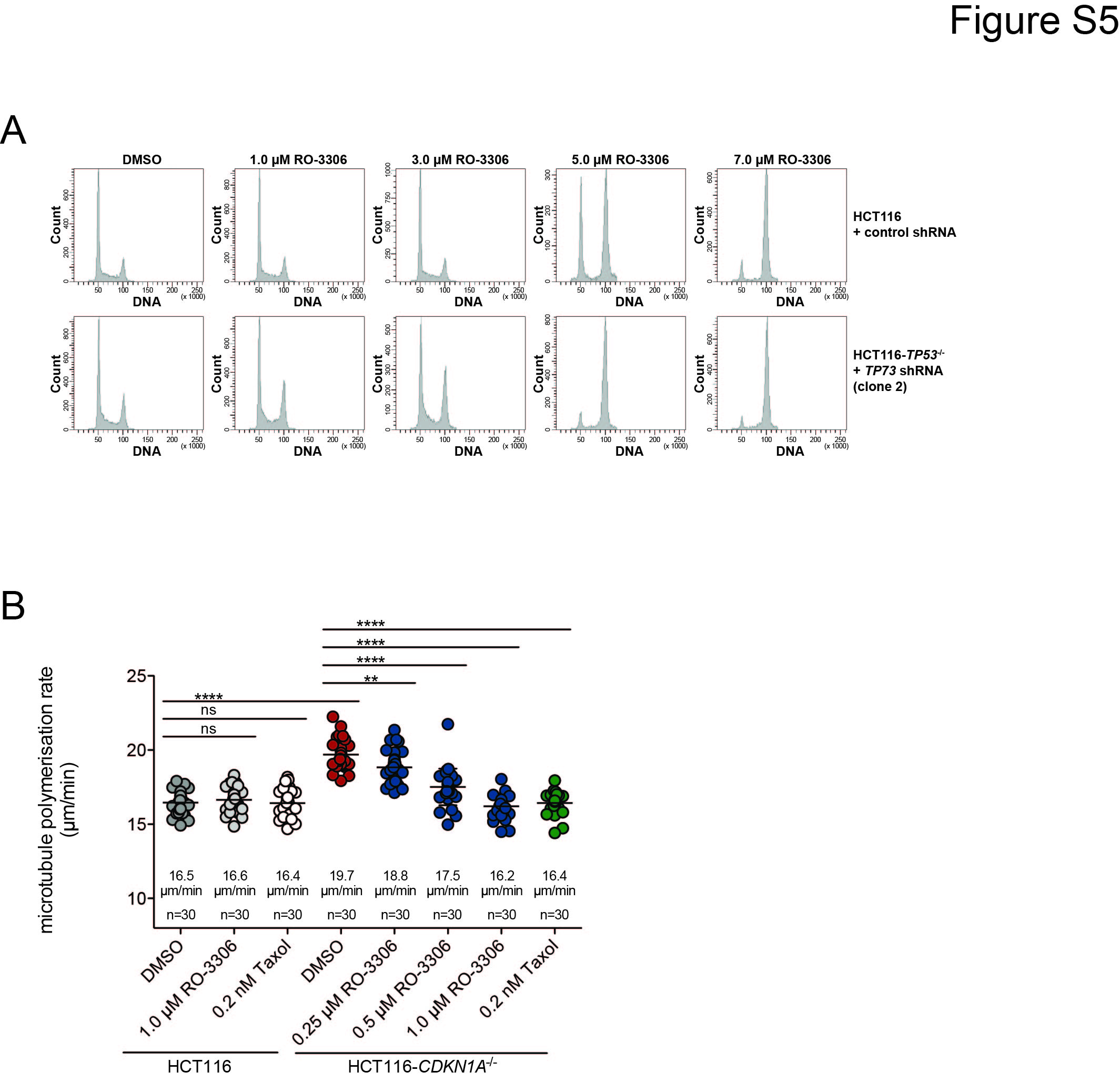

Supplement: Supplementary file 7 — Supplemental Figure S5 [file 41388_2020_1524_MOESM7_ESM.jpg]

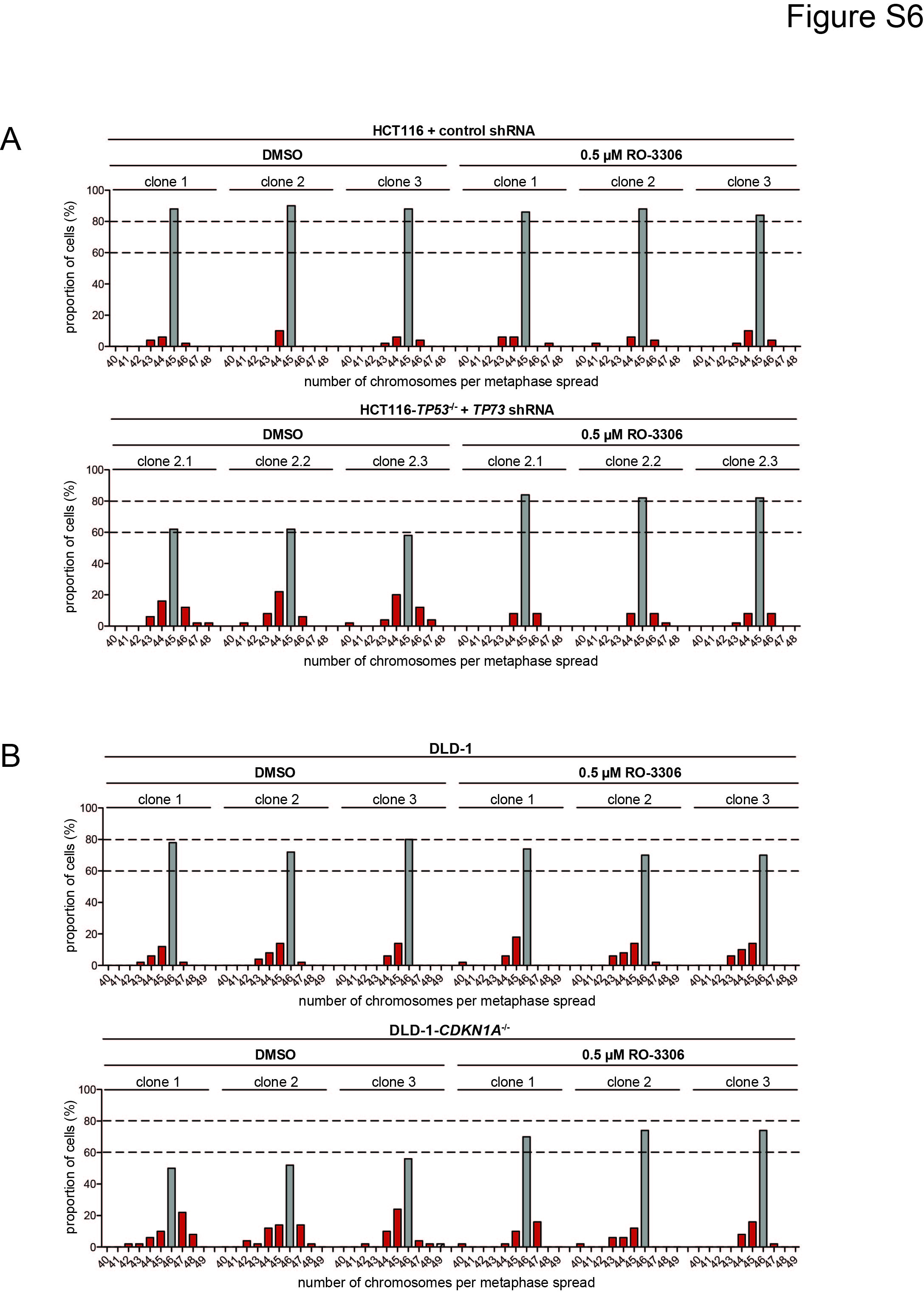

Supplement: Supplementary file 8 — Supplemental Figure S6 [file 41388_2020_1524_MOESM8_ESM.jpg]

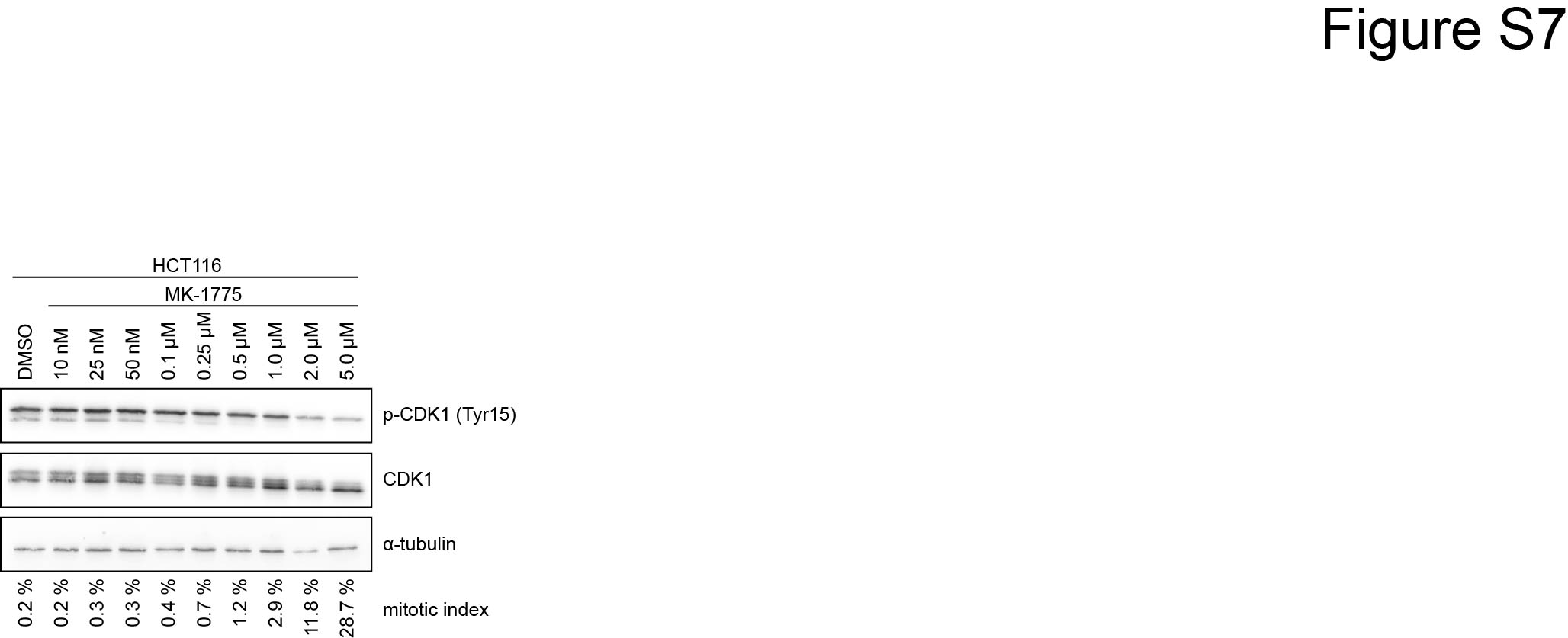

Supplement: Supplementary file 9 — Supplemental Figure S7 [file 41388_2020_1524_MOESM9_ESM.jpg]

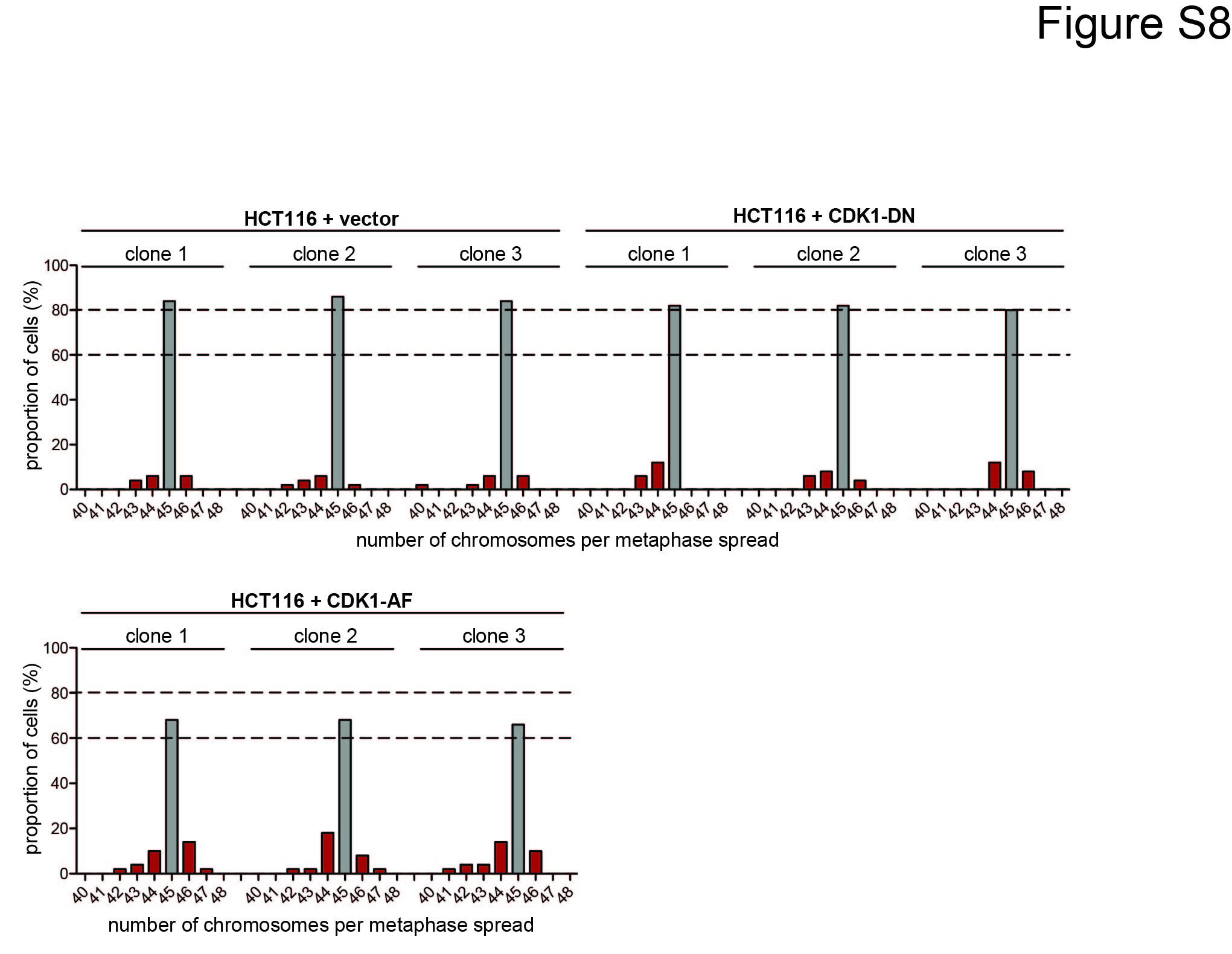

Supplement: Supplementary file 10 — Supplemental Figure S8 [file 41388_2020_1524_MOESM10_ESM.jpg]

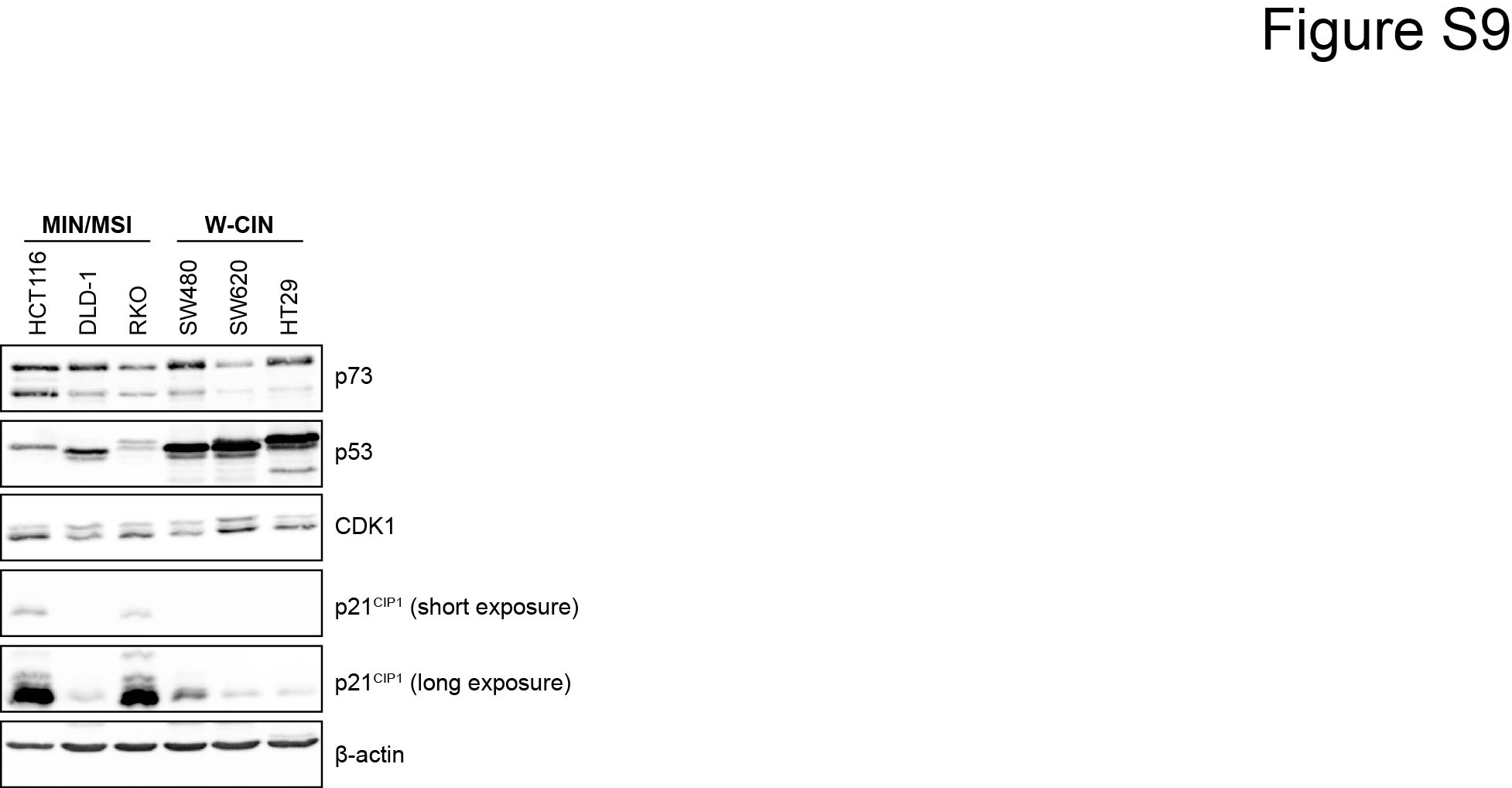

Supplement: Supplementary file 11 — Supplemental Figure S9 [file 41388_2020_1524_MOESM11_ESM.jpg]

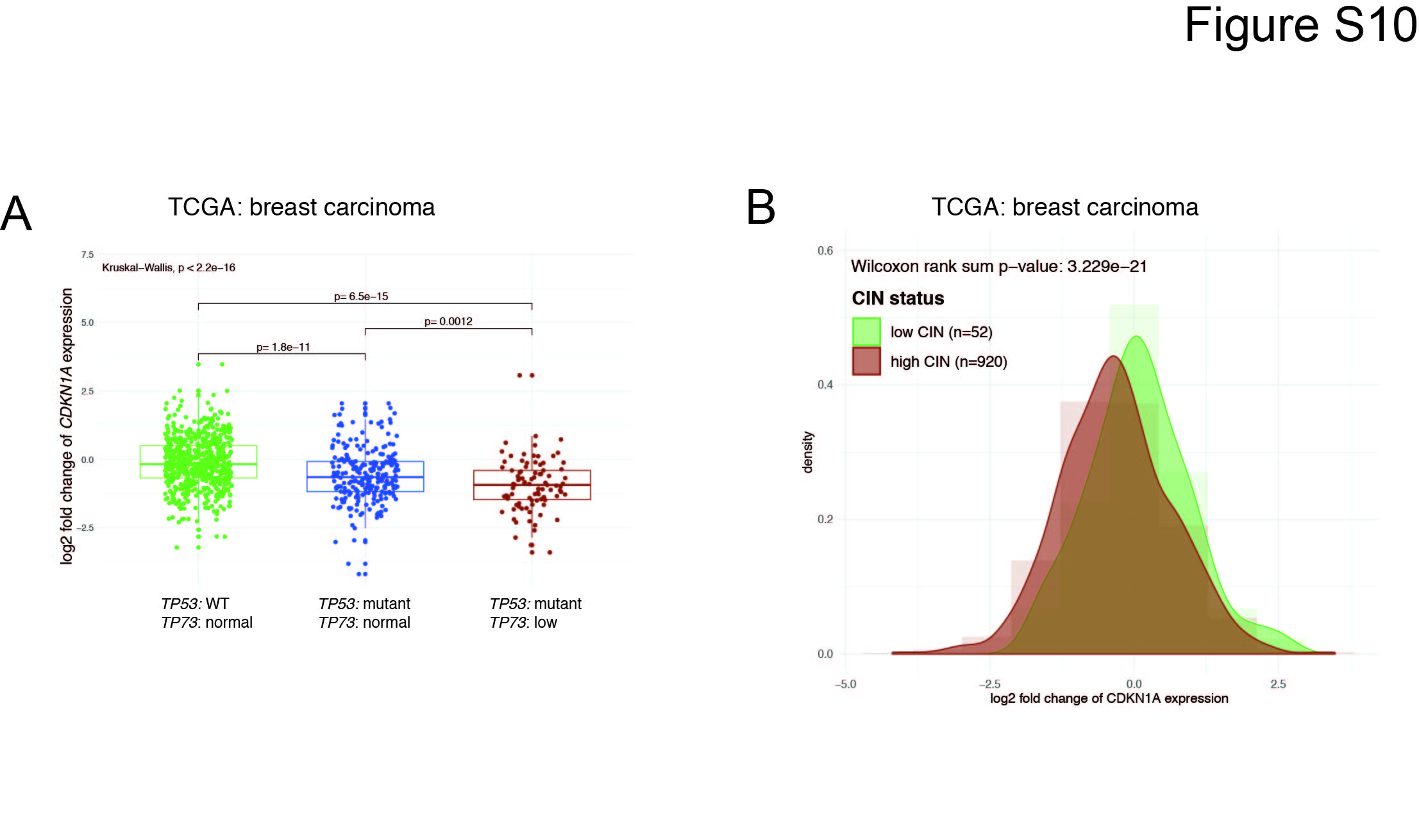

Supplement: Supplementary file 12 — Supplemental Figure S10 [file 41388_2020_1524_MOESM12_ESM.jpg]
